# Understanding the environmental and social risks from the international trade in ornamental plants

**DOI:** 10.1093/biosci/biae124

**Published:** 2025-01-17

**Authors:** Amy Hinsley, Alice C Hughes, Johan van Valkenburg, Tariq Stark, Jeroen van Delft, William Sutherland, Silviu O Petrovan

**Affiliations:** Department of Biology, University of Oxford, Oxford, England, United Kingdom; School of Biological Sciences, University of Hong Kong, Hong Kong, People's Republic of China; Netherlands Institute for Vectors, Invasive Plants, and Plant Health, Wageningen, Netherlands; Reptile, Amphibian, and Fish Conservation Netherlands, Nijmegen, Netherlands; Reptile, Amphibian, and Fish Conservation Netherlands and with the Netherlands Centre of Expertise on Exotic Species, Nijmegen, Netherlands; Department of Zoology and with BioRISC,, University of Cambridge, Cambridge, England, United Kingdom; Department of Zoology and with BioRISC,, University of Cambridge, Cambridge, England, United Kingdom

**Keywords:** global plant trade, pests and pathogens, food security, water security, sustainability

## Abstract

The multibillion dollar ornamental plant trade benefits economies worldwide, but shifting and rapidly expanding globalized supply chains have exacerbated complex environmental, sustainability, and biosecurity risks. We review the environmental and social risks of this international trade, complementing it with analyses of illegal trade seizures and plant contaminant interception data from the Netherlands and the United Kingdom. We show global increases in ornamental plant trade, with supply expansions in East Africa and South America, and highlight risks and impacts including biodiversity loss, aquifer depletion, pollution, undermined access and benefit sharing, and food security. Despite risk mitigation efforts, the interception data showed considerable volumes of contaminants in ornamental plant shipments, but taxonomic identification was not always possible, highlighting uncertainties in assessing biosecurity risks. With high-volume and fast-moving transit of ornamental plants around the world, it is essential that production standards are improved and that data on specific risks from trade are collected and shared to allow for mitigation.

In recent years, there has been a growing focus on the environmental consequences for exporter and importer nations of commodity exchange. These discussions have largely been focused on the impacts of trading food commodities, including their role in spreading invasive species (Paini et al. 2016), and pledges of zero-deforestation supply chains (Hughes 2023). However, although food and similar commodities have been encompassed in global targets such as the Kunming–Montreal Global Biodiversity Framework (i.e., supply chain tracking and zero-deforestation pathways have been included), the impacts of trade in inessential commodities, such as flowers, has generally been neglected and overlooked (Lenzen et al. 2012). This is despite the fact that these products are important global commodities, which, in 2022, had an export value of US$10 billion for cut flowers and foliage, and US$13 billion for live plants and bulbs (figure 1; International Trade Centre 2024). Furthermore, the ornamental plant sector has been growing at a steady rate in recent years and is highly mutable, with frequent changes observed in demand for different products, the emergence of supply countries such as Colombia, Ecuador, Kenya and Ethiopia (figure 2), and shifting trade practices including the movement to vegetative over seed propagation (Hammond et al. 2023). Such dynamic supply chains can present significant challenges for the quantification and management of risks, including those related to biosecurity and illegal wild plant trade, as well as broader environmental impacts.

**Figure 1.**
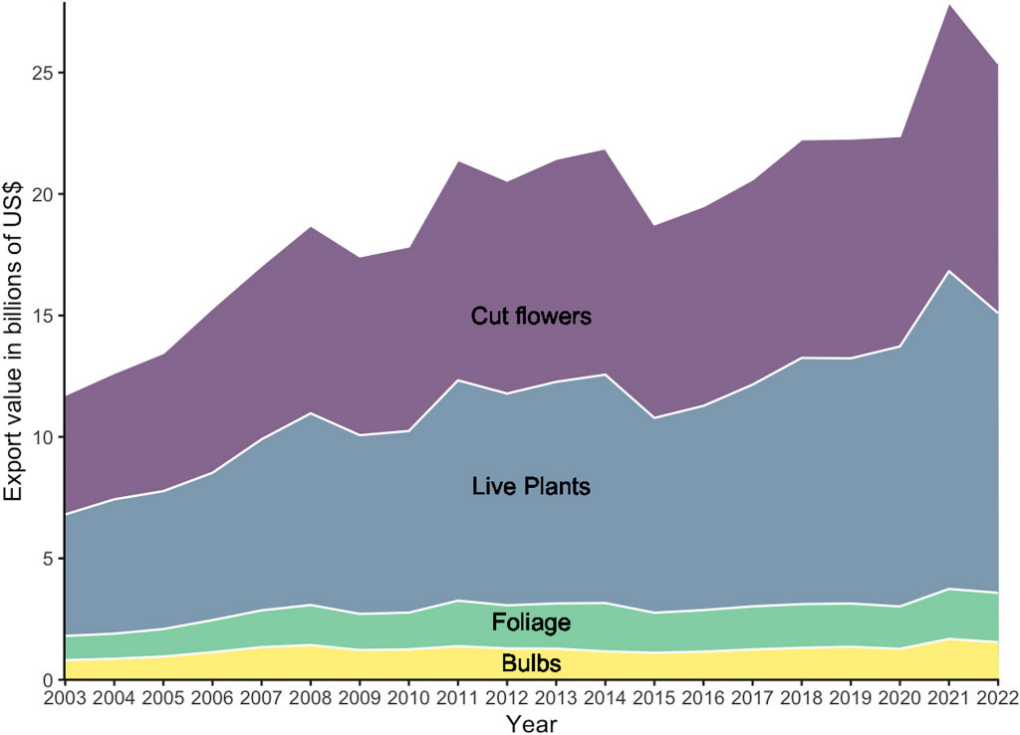
Annual global export value of ornamental plants between 2003 and 2022, showing data from the HS codes for cut flowers (HS0603), live plants (HS0602), bulbs (HS0601), and ornamental foliage (HS0604) based on data downloaded in July 2024 (International Trade Centre 2024).

**Figure 2.**
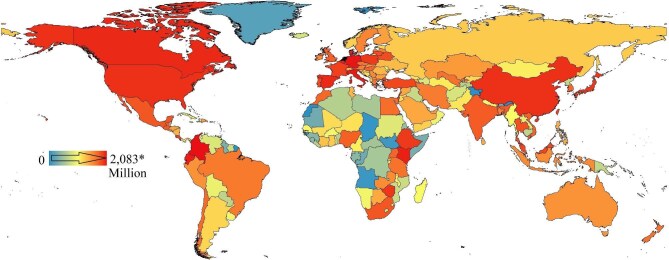
Maximum annual export value of ornamental plants (2000–2022) in US dollars. The Netherlands exported the most, at US$12.7 billion; this is not noted on the figure legend because it is disproportionately higher than any other region, although this includes reexported plants. Colombia, Ecuador, and Kenya have become major floricultural exporters. Source: The data are from UN COMTRADE.

To address this point, we bring together research into environmental, biodiversity, and social impacts of the ornamental plant trade and make the case for greater attention to the management of this increasingly important international trade. Although there has been research into specific threats that may be associated with supply chains for some commodities, this has rarely included ornamental plants outside a few specific countries (Hughes 2001, Sæthre et al. 2010), and, to our knowledge, there has not been a synthesis of the diverse studies examining the range of impacts for this trade. We discuss the current known and emerging risks to both importers and exporters of ornamental plants, including cultivated plants (which constitute the vast majority in trade by volume) but also wild-harvested ornamental plants. To address some of the gaps in knowledge, we analyze data from publicly available data sets on the incidence of ornamental plant illegal trade seizures. We also add novel data sets of contaminant interceptions and vertebrate hitchhikers found in plant shipments, often reported by members of the public and not normally collated in any public database. We synthesize the existing literature and these analyses to give a more complete picture of the true risks and impacts of the ornamental plant trade and explore potential approaches to address them.

## Changing patterns of trade

**Figure 3.**
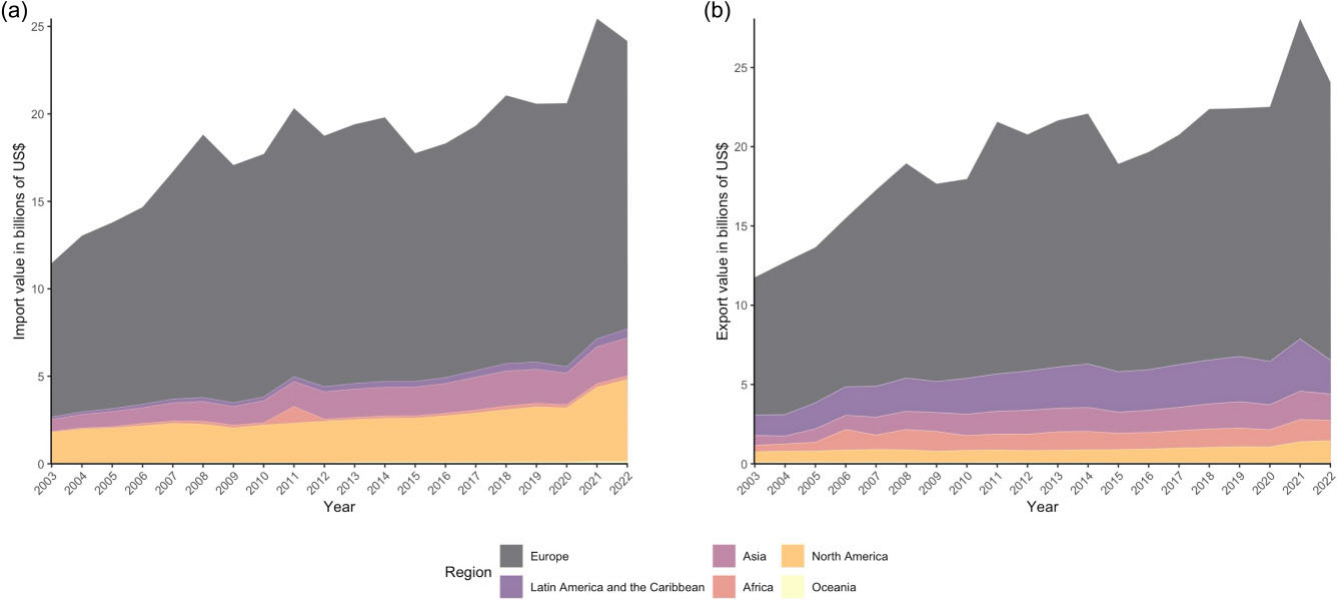
Values of ornamental plant imports (a) and exports (b) reported by each region between 2003 and 2022 as reported in the TradeMap database (International Trade Centre 2023).

**Figure 4.**
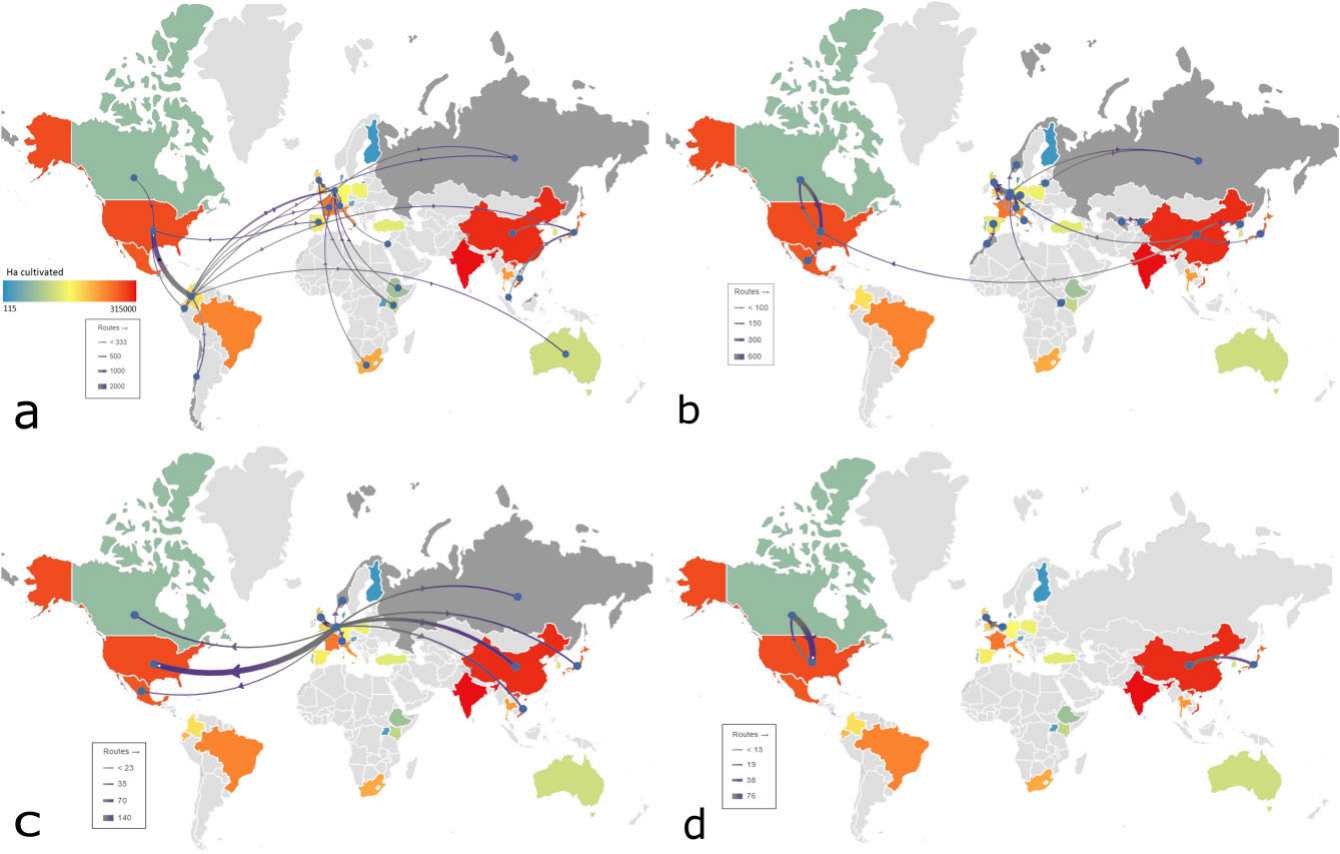
The trade of live ornamental plants and cut flowers and foliage between nations and cultivation of large scale floriculture in hectares (the color ramp) in 2020. (a) Cut flowers. (b) Live ornamental plants. (c) Bulbs. (d) Cut foliage. Source: The data are from Rabobank 2022. The gray end of arrows indicates export, and the arrow heads also indicate trade direction.

Ornamental plant trade pathways are constantly changing because of economic, logistical, and geopolitical reasons (e.g., Brexit, changing tariffs); significant industry growth in the last two decades (figure 1); and the diversification of source markets (figures 2, 3, and 4). The scale of both the production and the diversity of trade should not be underestimated, with varying degrees of production across much of the globe (figure 3) and specialism in different products showing different spatial patterns (figure 4). Cut flowers currently predominantly come from Latin America and parts of Africa, largely for use in North America and Europe. Live (potted) ornamental plants have less complex trading patterns but an added risk from any imported potting medium and may be less prone to reexport than other classes of ornamental plants, although China provides a significant hub (figure 4). For bulbs, the Netherlands dominates global trade as an exporter, whereas for foliage, there are more limited markets (Canada–United States, Netherlands–United Kingdom, China–Japan; figure 4). Changing markets, such as increased demand for orchids in countries including Vietnam (Yuan et al. 2021), may also change the patterns of risk, especially if the new exporting or importing countries have not yet developed rigorous phytosanitary procedures and capacities. These dynamic global trade patterns (figures 3 and 4) provide opportunities to transport and transfer pests and pathogens, as well as various chemical residues, because of different legislation between administrative areas (e.g., many chemicals banned in the EU are imported as residues on the surfaces of ornamental plants; Toumi et al. 2017). Although some aspects of the trade are reflective of overall patterns in trade globalization, ornamental plants are different from other biological commodities in trade, such as food, in being of relatively low unit value and of typically no nutritional value, and they can be extremely short lived in the case of cut flowers and foliage. They may therefore be more reflective of changing patterns of wealth and will then continue to shift as the economic profiles of regions change and new markets develop with growing affluence (Button 2020). In addition, changes in ornamental plant consumer preferences and trends in gardening mean that new trading links and markets can rapidly become established, some with substantially different associated risks. For example, the growing ornamental market for old or even ancient (i.e., 80 to more than 100 or even more than 500 years old) olive trees *Olea europaea* (see box 1) involves winter uprooting and transport of farmland trees with numerous trunk hollows and very large root balls, both of which can host and therefore transport a wide range of reptiles and their eggs (Silva-Rocha et al. 2015), insects, and arachnids (Bellvert and Arnedo 2016, Sherwood 2022). The demand for other exotic trees, palms, and shrubs is also high (Beaury et al. 2021) and can function as a vector for introductions (Adamopoulou and Pafilis 2019, Rebelo et al. 2019). These factors combine to create specific challenges for trade sustainability and logistics but also for ensuring that such shipments are free of contaminants, in particular if the trade expands in regions that require long distance, intercontinental shipments.

Box 1. **Illegal trade in ornamental plants.**

Data on any illegal trade is difficult to collect because of its covert and sensitive nature, but illegal plant trade is especially difficult to interpret, because of its low priority for those involved in enforcement and policy (Margulies et al. 2019). We downloaded all seizures related to plants from 2013–2022 from the TRAFFIC Wildlife Trade Data Portal (www.wildlifetradeportal.org). For the 3336 incidents reported, we removed those referring to timber, medicinal, and edible products, identified manually using the descriptive text for each incident. The remaining 104 incidents were linked to a seizure or illegal harvesting event for ornamental plants, involving at least 109,180 individual plants or seeds. Of these seizures, 59 incidents involving at least 69,589 individual plants or seeds had details that suggested the trade was international. We summarized key trade routes and taxa but note that these are taken from only one source of publicly available data on seizures. Furthermore, they represent evidence of individual seizures rather than of broader patterns of trade, because of biases linked to enforcement effort, taxonomic priorities, and difficulties in plant identification.

The reported illegal trade incidents primarily included seizures of Cactaceae (*n* = 27, with 16 named genera, including *Ariocarpus, Astrophytum*, and *Mammillaria*) and Orchidaceae (*n* = 14), with six named genera including *Dendrobium* and *Bulbophyllum*, with nine incidents involving seizures of various succulents (four unnamed succulents; two *Dudleya* species; one each *Conophytum, Aloe*, and *Euphorbia*), three carnivorous plants (three *Nepenthes* and one *Dionaea* species), and one each involving water lilies (*Nymphaea* spp.), cycads (*Cycas* spp.), and unnamed aquatic plants. The seizures occurred in 26 countries, with the highest numbers in Poland (*n* = 10), Belgium (*n* = 9), China (*n* = 8), Sweden (*n* = 7), and Malta (*n* = 7).

The total seizure volume between 2013 and 2022 was dominated by substantial individual seizures of the succulent taxa, including 60,397 *Conophytum* plants seized from Korean nationals in South Africa in 2020, 3715 *Dudleya* plants seized from Korean nationals in the United States in 2018, and 1100 *Aloe ferox* plants seized in the United Kingdom but originating in South Africa. However, many taxa, including orchids, are hard to identify at species level and can therefore be laundered in shipments of legally traded plants, meaning that these numbers are likely to be underestimates of true trade (Hinsley et al. 2017). Such inclusions of wild-harvested plants inherently also increase the biosecurity risk of pathogens and pests (Hulme et al. 2018).

International trade can occur as a consequence of geographic specialization related to natural resource availability, the production of specific goods at reduced costs (such as because of cheaper labor or production costs), or less stringent environmental legislation in the producing country. Global trade has increased significantly in recent decades, including for products such as fresh vegetables and ornamental plants that have become year-round imports into Europe from sub-Saharan Africa, rather than reflecting seasonal patterns (figures 1–3; Dolan and Humphrey 2000, Darras 2020a). The form of commodity, as well as the trade route, has different implications and impacts; for example, the Netherlands is a key country for ornamental plant trade, including reexports (figure 4), therefore potentially magnifying risks to exchange of pests, pathogens and other hitchhikers between shipments. In 2022, the Netherlands was the top exporter (by value) of all ornamental plant products, with other European countries including Germany and Italy being the top exporters for all such products except cut flowers (table 1, figure 2). The United States, Germany, and the Netherlands were in the top three importers for all ornamental plant products except bulbs, whereas China was the third highest value importer (table 1). These different forms of commodity have different types of impacts, because of both the different abilities to host pests or invaders and the very short storage time (with cut flowers hosting the highest risk of invasive species; figure 3; Australian Department of Agriculture 2019, Netherlands Food and Consumer Product Safety Authority 2020). Furthermore, cut flowers require the fastest transport, sometimes including low-temperature and even pressurized environments and generate more waste than potted plants and bulbs do, which are less perishable and may have much lower carbon footprints (Swinn 2017). Critically, in relation to assessing risks and impacts, the geographical trade patterns of ornamental plants have changed over time, with the exports values from some regions doubling in global trade in recent decades despite the ongoing dominance of European export markets (figure 3).

**Table 1.** Top importers and exporters in 2022 for different ornamental plant products by their value in US dollars, as was reported in the TradeMap database (International Trade Centre 2023).

		**Top importers by value**		**Top exporters by value Exporters**	
**Product**	**HS code**	**Importer**	**Value (in millions of US dollars)**	**Exporter**	**Value (in millions of US dollars)**
Bulbs	0601	Germany	223.9	The Netherlands	1631.2
		United States	223.3	Germany	73.3
		China	139.4	Belgium	50.1
Live plants	0602	Germany	1483.2	The Netherlands	5323.6
		United States	957.5	Italy	950.5
		The Netherlands	924.1	Germany	828.6
Cut flowers	0603	United States	2464.3	The Netherlands	4758.2
		Germany	1311.6	Ecuador	1017.8
		The Netherlands	1084.6	Kenya	628.6
Ornamental foliage	0604	The Netherlands	399.8	The Netherlands	402.9
		United States	305.0	Italy	183.0
		Germany	167.2	Denmark	150.1

## Impacts on producers and exporters

Impacts of ornamental plant trade vary between producers, transit, and importers. Understanding these facets is crucial to manage where risks exist.

### Impacts on wild plant species in exporter regions

The impacts of harvest and trade on wild plants can be difficult to ascertain, partly because of plants receiving less attention from the public, conservation researchers, and policy-makers (Margulies et al. 2019). The commercial wild harvest of ornamental plants can be sustainable in some cases but must be carefully managed, with engagement throughout the whole supply chain (FFI 2018). However, many ornamental plants are poorly studied in the wild, making the estimation of sustainable harvest levels difficult using traditional approaches (Ticktin et al. 2023). For example, in South Africa, the collection of wild fynbos flowers used for bouquets has been regulated using quotas to prevent unsustainable harvest, accompanied by monitoring of wild populations and offtake levels (Bek et al. 2017, Privett et al. 2020). However, even in this established case, it has been difficult to maintain operations, because the profits from trade were insufficient to provide a stable income to support sustainable businesses in the long term (FFI 2018).

Besides legal trade, the illegal trade of ornamental plants for both domestic and international markets is known to be occurring on a substantial scale in multiple wild taxa, including orchids (Hinsley et al. 2017), cacti (Goettsch et al. 2015), and succulents (Margulies et al. 2023). Although the full impacts of this trade on wild populations are often undocumented, the ornamental plant trade is reported to have caused local extinctions of slipper orchids and extinctions in the wild of several cycad species harvested for the live plant trade, as well as Sprenger's tulip *Tulipa sprengeri*, and the Chilean blue crocus *Tecophilaea cyanocrocus*, harvested for the bulb trade (Maunder et al. 2001, Hinsley et al. 2023). However, trade-related extinctions or declines of plants are probably underreported because of a lack of research on which species are being harvested for trade (Margulies et al. 2019) and on the impacts of trade threats on wild populations (Hinsley et al. 2023). It is likely that there are further undocumented extinctions, because harvesters of high-value taxa such as slipper orchids are known to strip entire habitats of plants immediately after discovering them (Averyanov et al. 2014).

Although wild harvest plays a role in international trade, for species listed in the appendices of the Convention on the International Trade in Endangered Species of Wild Fauna and Flora (CITES), cultivated plants now make up the vast majority of exports, showing a shift from wild sourced to cultivated plants in most plant orders (Harfoot et al. 2018). This includes more than 99.9% of live orchids legally commercially traded between 1996 and 2015 and reported as artificially propagated (Hinsley et al. 2017). Cultivation is often promoted as a solution to reduce unsustainable wild harvest, but it does not automatically remove pressure on wild-harvested plants (Williams et al. 2014). There are a range of factors that must be considered to assess the potential impact of introducing cultivated products to a market, including consumer preferences and the relative cost of cultivation compared to wild harvest (Phelps et al. 2014). Where wild ornamental plants are easily accessed or cheaper than their cultivated alternatives, wild specimens are often still sold after cultivation has been introduced, even being found alongside their cultivated alternatives in domestic Southeast Asian and Chinese orchid markets (Phelps et al. 2014, Gale et al. 2019). Furthermore, for taxa such as *Paphiopedilum* orchids, where wild-sourced commercial trade is prohibited, legal shipments of cultivated stock can allow wild plants to be laundered into international trade (Hinsley et al. 2016). In addition, specialist markets for rare species can also drive wild harvest because of preferences for rare plants or newly discovered species (Hinsley et al. 2016). Cultivation may rely in some part on wild harvested mother stock, resulting in either whole plants or seeds being removed from the wild to sustain nursery stock (Liu et al. 2019) or to introduce new varieties or forms to the trade. Indeed, although the introduction of cultivation can lead to a decrease in harvesting in some cases, it is not inherently associated with a reduction in conservation threat (Liu et al. 2019), and it may also increase wild harvest, as in the case of the harvest of ornamental foliage from the xaté palm (*Chamaedorea ernesti-augusti*; see box 2; Williams et al. 2014).

Box 2. **Invertebrate pests detected in ornamental plant shipments in the Netherlands and United Kingdom.**

There is no comprehensive international data set on the types and numbers of pests that are intercepted during checks in the ornamental trade. We were provided with data by customs biosecurity officials in the Netherlands to access a database of over 8000 contaminant interceptions in 2017–2018 (Pieters et al. 2018), and in the United Kingdom to obtain records from 2021–2023 from the public, aggregated data DEFRA portal (https://planthealthportal.defra.gov.uk/trade/imports/non-compliance). We analyzed these to provide a snapshot of the types of pests that were detected, defining pests as those mentioned in official databases and publications as having negative impacts on agricultural crops, humans (e.g., as nuisance) or biodiversity. Relevant data to support analyses, and full methods, are available in supplemental file S2.

In 2017, the Netherlands imported 14,999 tons of bulbs, 362,654 tons of live plants, 272,798 tons of cut flowers, and 84,714 tons of ornamental foliage (International Trade Centre 2023). In this time, 1538 interceptions of 459 different pest types were detected, with the most frequently intercepted pest *Trialeurodes vaporarium*, the glasshouse whitefly (*n* = 193), followed by the moth *Helicoverpa armigera* (*n* = 81). In 2021–2023, the United Kingdom reported 481 interceptions of 36 notifiable pest types, with *Bemisia tabaci*, the silverleaf whitefly, the most frequently intercepted (*n* = 277 interceptions), followed by *Liriomyza* spp. (*n* = 48; see supplemental file S2, figure S1).

The majority of interceptions in both data sets were in the class Insecta (Netherlands, *n* = 1394, 90.6% of all interceptions; United Kingdom, *n* = 390, 81.1%). The Netherlands also intercepted 97 pests in the class Arachnida (6.3%), and 11 each in Gastropoda and Collembola (0.7% each), with smaller numbers in eight other classes. After insects, the United Kingdom had 36 interceptions in the class Xanthomodales (bacterial phytopathogens; 7.5%), 21 in Peronosporea (4.4%), and 12 in Sodariomycetes (2.5%), with smaller numbers in six other classes. Although the majority of interceptions in both data sets were identified to at least the species level (United Kingdom, 403, 83.8% of the total; Netherlands, 1089, 70.8% of the total), some were identified only at higher taxonomic levels, including to extremely diverse groups that included numerous serious pest species. In the Netherlands data, this included one interception reported at Phylum level (Arthropoda), 16 at class (Collembola, 8; Insecta, 4; Gastropoda, 2; Chilopoda and Arachnida, 1 each), and 82 at order (e.g., Lepidoptera, 28), whereas the United Kingdom data included one interception reported as the order Thysanoptera. Given the difficulty of checking large volumes of potted plants and cut flowers for invertebrate, fungal, and microorganism contaminants, it is likely that these interceptions represent a small fraction of the total contaminants in these shipments.

**Figure 5.**
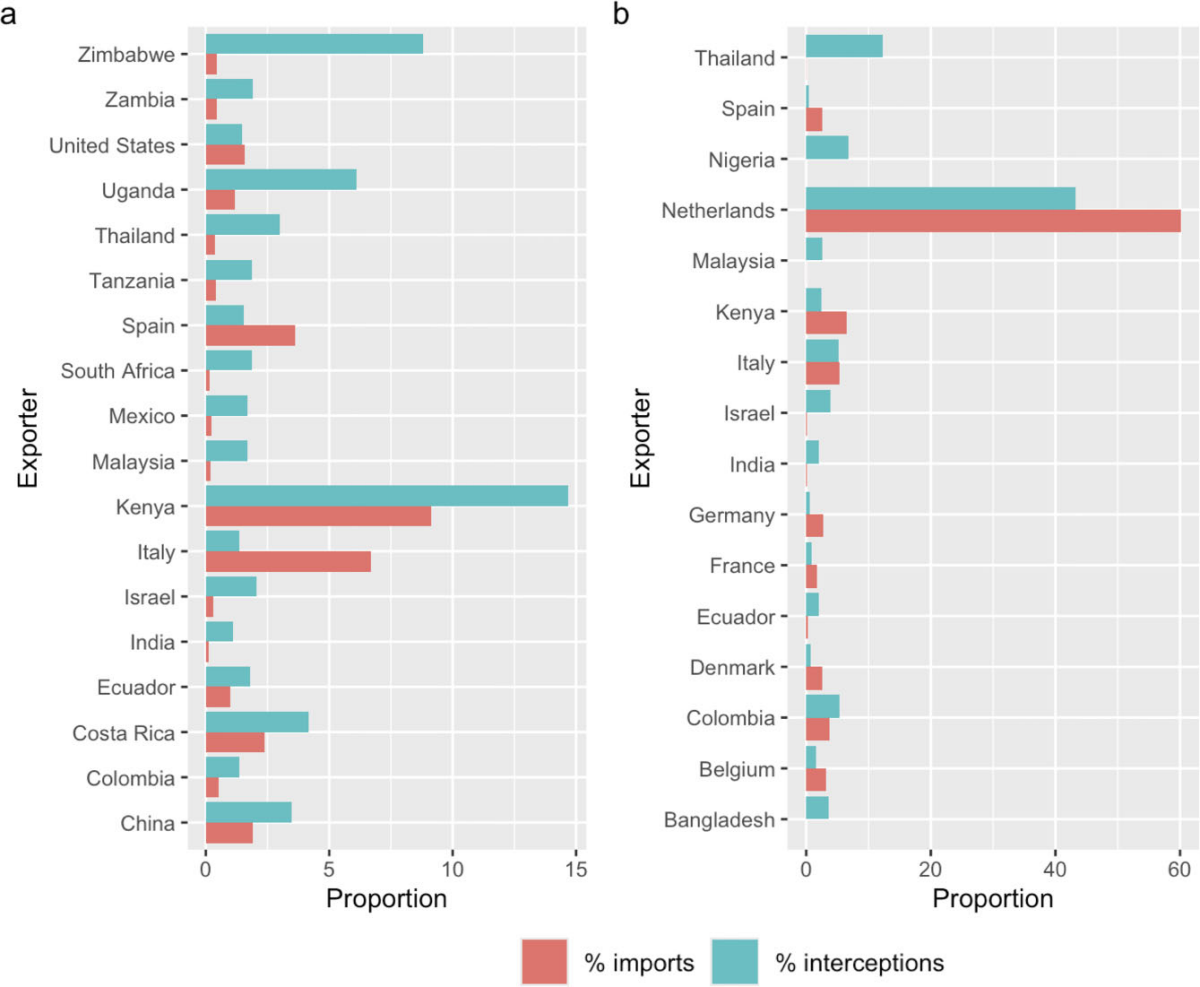
Proportion of (a) total pest interceptions in ornamental plant imports to the Netherlands from different exporters in 2017, compared with the proportion of all imports to the Netherlands in 2017 as reported in the HS customs data, and (b) regulated pest interceptions to the United Kingdom between 2021 and 2022, showing only exporters with more than 1% of all pest interceptions or imports.

The top taxon–country combinations for interception of notifiable pests to the United Kingdom in 2021–2023 were *Solanum* from the Netherlands and *Chrysanthemum* from Colombia, whereas roses from both Kenya and Uganda contained the highest numbers of pests in the Netherlands 2017 data (see supplemental file S2). Comparing the proportion of total interceptions from each country with the proportion of imports from those countries to the Netherlands in 2017 and to the United Kingdom in 2021–2022 (the last year when all import data were available) shows that imports from some countries had a disproportionate probability of harboring pests. We performed a Fisher's exact test comparing all imports with interceptions in that year, restricting the analysis to any country with nonzero values for both imports and interceptions and more than 1% of the total for either category. We found that seven countries had significantly fewer interceptions than would be expected on the basis of the proportion of total imports from that country (Belgium, Denmark, Germany, France, Ethiopia, Italy, Spain). Fifteen countries had significantly more interceptions than would be expected, including exporters with growing market shares, such as Ecuador, Colombia, and Kenya (figure 5a). In the same analysis for the United Kingdom in 2021–2022, six countries had significantly fewer pests than would be expected on the basis of proportion of imports (Spain, Germany, Denmark, Kenya, Belgium, the Netherlands), and five had significantly more pest interceptions that would be expected (Ecuador, India, Israel, Malaysia, Thailand; figure 5b). The reasons for these discrepancies are difficult to ascertain, but higher detections of contaminants from certain countries can lead to higher search effort in subsequent shipments from that country, meaning such data are difficult to interpret, and shifting standards make understanding genuine patterns of risk hard to quantify.

### Impact on water access and pollution

Water use in the horticultural industry is a major and growing issue. Globally, agriculture and horticulture represent 70% of freshwater withdrawals, and by 2030, a 40% shortfall is estimated between demand and supply (UNEP 2015, FAO 2017a, Mazzucato et al. 2023). These withdrawals can be associated not only with water depletion but also with soil salinization and water eutrophication (Wolosin 2006). Water demands for ornamental plant production can be substantial in some countries; for example, in India, floriculture was estimated to require about twenty times the water that cotton requires to be grown for the same area and even more relative to rice and other food crops (Sharma 2008). Such issues should not be overlooked, especially given the large and increasing areas under floriculture (figure 4).

Kenya is the sixth largest supplier of cut flowers to the international market (US$852 million per year, 13.5% of international export outside Europe and North America; figure 4; OEC 2023a). The floriculture industry in Kenya is responsible for up to 98% of water abstraction from major lakes, such as lake Naivasha (Mekonnen and Hoekstra 2014, Kameri-Mbote and Odhiambo 2015). High water usage for floriculture can affect water access, may cause aquifers to drop, and can affect other livelihoods (such as fishing) because of high levels of water pollution (Gudeta 2012, Hatch and Wells 2012, Sisay et al. 2017, Özcerkes 2018). Furthermore, because regions such as East Africa are particularly vulnerable to climatic change (see figure 2), with most crops for local consumption dependent on rainwater, the depletion of aquifers may exacerbate the risks of food insecurity resulting from climate change, as well as increase vulnerability to drought (Marigi 2017, Ahmadalipour and Moradkhani 2018, Ahmadalipour et al. 2019). High water use can have lasting effects in regions with expansive floriculture and has been linked to aquifer depletion, which is especially problematic in regions with increased frequency of drought (Haile et al. 2020, Lottering et al. 2021). Furthermore, these increases in water use are in addition to necessary and projected increases in irrigation and water use for agriculture in developing economies, where much floriculture occurs (Hughes 2001, FAO 2011, 2021). Many African countries currently leading in floriculture show some of the highest potential for local conflict risk associated with water stress globally, meaning that the high water requirements of floriculture have ramifications for future political stability (World Water Atlas 2023). Water availability remains one of the major criteria for assessment of the viability and development of a floricultural industry, but calculations of future need may fail to account for other national water requirements (Kerkhoven et al. 2014).

Similarly, in Ecuador, which has become one of the top five flower exporters in the world (Guaita-Pradas et al. 2023), this industry is one of the most dynamic areas of the economy, supporting a substantial proportion of national exports and much-needed rural employment, especially employing women (Newman 2002, Raynolds 2012). Floriculture can show both long- and short-term competition with food production, and food security and water scarcity have become increasingly problematic as a consequence of the heavy water use for flower production in the country (Mena-Vásconez et al. 2016). These concerns have remained even after the 2008 constitution and a 2014 water law prioritizing the production of food over the production of flowers in Ecuador, primarily because smallholder producers cannot garner protection from the new regulations (Mena-Vásconez et al. 2016). In 2021, Ecuador represented 3.7% of global cut flower export, at around US$979 million annually (OEC 2023a). Floriculture remains an important industry within Ecuador, and although regulations have been implemented to try to reduce the negative implications for local regions and communities, such as overusing water, and the use of the best agricultural land for flower production, more work is clearly needed (Mena-Vásconez et al. 2016).

In addition, although flowers are regarded as agricultural imports and therefore must be pest free and free of pesticide residues in EU import standards, they are typically not food and can therefore have significant pesticide residues present, because most countries do not have limits on pesticides in flowers not intended for consumption (Pereira et al. 2021). Some of the pesticides used in both developing and developed economies can have severe health side effects in those handling and packaging the flowers, as well on the watercourses in the regions they are grown in (Munnia et al. 1999, Tenenbaum 2002, Lu 2005, Toumi et al. 2016, Gooijer et al. 2019). Pesticides from floriculture have been linked to poisoning in supply countries, as well as neurological impacts in children and adults (Tenenbaum 2002, Grandjean et al. 2006, Donahoe 2008, González-Andrade et al. 2010, Tsimbiri et al. 2015, Mrema et al. 2017, Suarez-Lopez et al. 2017, Pereira et al. 2021). These health issues have been known for decades, with some major issues noted (Donahue 2008). In total, on the basis of a GHS standard (Globally Harmonized System of Classification and Labelling of Chemicals) 201 pesticides used in floriculture are noted as “can pose critical risks to humans,” including in some cases various carcinogens, compounds fatal on inhalation, affecting reproductive and respiratory systems and even causing mutagenesis (Pereira et al. 2021). Chemicals from floriculture, including persistent organochlorines, DDT (dichlorodiphenyltrichloroethane), aldrin, and dieldrin, may be directly discharged into water courses (Pereira et al. 2021). In addition, some have significant environmental effects, including the lasting impacts of DDT on birds (Bouwman et al. 2019). Moreover, although many of these chemicals have been banned in the United States and Europe, they remain in use in developing economies, and may even be dumped in large quantities when listed as “obsolete” with widespread impacts on the environment and local human populations (Mengistie 2016, Negatu et al. 2021, Rajak et al. 2023). Flower cultivation often uses high pesticide application, with 201 compounds identified in a global review, 93 of which were banned in the European Union (Pereira et al. 2021, Bär et al. 2022). This affects both supplier and importer countries, because high pesticide loads may remain on the plants. Almost 70% of the water samples in water bodies in the vicinity of floriculture production areas were contaminated with pesticides in a global review (Pereira et al. 2021), and many industries lack adequate regulations to reduce local impacts on people or the environment (Merga et al. 2021, Gelaye 2023). Furthermore, around 25% of the pesticides sprayed in greenhouses reach the soil, where they can reduce soil metabolic rate and total soil biomass (Querejeta et al. 2012, Pereira et al. 2021). As a separate issue, plastic mulching and the application of compost with high plastic contamination to grow both vegetables and ornamental plants are widely practiced (Wittwer 1993, Jiao et al. 2022). Both practices add considerable amounts of microplastics into the environment, which contaminates the potting substrates in plant nurseries. The scope of this problem is largely unrecognized. Monitoring ecological effects and legislation on the basis of maximum thresholds is needed to ensure that microplastic concentration remains at “safe” levels (van Schothorst et al. 2021).

Furthermore, highly perishable commodities, such as cut flowers, have high waste during growth; for example, in 1994, it was estimated that 1 hectare of roses and 1 hectare of carnations produced 40 kilograms (kg) and 150 kg of waste per month, respectively (figure 4; Barriga 2006). Also, cut flowers and foliage have shorter shelf lives than live plants and bulbs do, resulting in high waste at the retailer; for example, in the United Kingdom alone, an estimated 34,700 tons of cut flowers were used and ended up in waste streams (such as landfills) each year (Petrou and Iacovidou 2015). Clearly, better methods, such as composting for cut flower waste, are warranted, as are better supply chain management techniques to better predict supply and demand to reduce wastage.

Floriculture standards have improved over the last two decades, including the introductions of certification schemes (Raynolds 2012), aimed at improving workers conditions and reducing environmental impacts. However, a lack of monitoring in some regions means that production standards can still be highly variable (Leipold and Morgante 2013), as can the working conditions for those involved (Wareru 2022). Therefore, although ornamental plant production is unquestionably economically and socially important in many regions, better standards are needed to guarantee local rights and to reduce harmful local impacts on people, the environment, and wild species. Such methods can be implemented but would need to be added to import requirements and can include stronger use of private voluntary standards, such as improved standards compliance in regional and global value chains (Wainwright et al. 2014, Krishnan 2018).

### Wider environmental and social impacts

Producing any agricultural product for export has impacts beyond biodiversity and water use, including other important environmental and social impacts that may be overlooked. For example, the need for low-temperature transport of short-lived products between continents means that the cut flower trade is responsible for a carbon footprint estimated to be as high as 3 kg of carbon dioxide per flower and up to 32.3 kg per bouquet (Lansink and Bezlepkin 2003, Williams 2007, Berners-Lee 2020) and also contributes other emissions, such as sulfur dioxide (Lan et al. 2022). The climate related impacts include the energy cost during production, including greenhouse ventilation, heating and cooling, irrigation, and fertilizer use, and during transportation (Darras 2020b), especially given that most flowers (with a few exceptions, such as snapdragons) require refrigeration, which may exceed 120 hours for certain flowers (such as roses and gypsophila; Swinn 2017). Transitioning toward renewable energy will, in time, decrease these impacts, although that is harder to achieve in relation to long-distance air haulage, and such infrastructure may be challenging to establish in some producer countries. Potted plants and especially flower bulbs are less perishable (have longer shelf lives) and involve a lower carbon footprint and therefore might represent better options.

An important issue to consider for potted plants in terms of their carbon and environmental footprint is the potting medium, especially peat (Alexander et al. 2008). Peatlands are highly threatened palustrine wetlands, but peat has been a major ingredient for horticulture and potting mediums, at the expense of bogs, marshes, and other habitats where peat can be extracted (Alexander et al. 2008). The continued extraction of peat in often already degraded peatlands has led to a decreased ability for peat lands to act as carbon and nitrogen sinks and to store water, and, because of habitat destruction and degradation, this led to a significant loss of biodiversity in these already threatened habitats (Joosten 2015). Potting mediums that lack peat are widely available, and their use may reduce these impacts.

An often overlooked issue is food security, which can be reduced via land grabbing or soil and water pollution (Tadele 2009, Wainwright et al. 2014, Kirigia et al. 2016). Furthermore, regions can become especially vulnerable because of changing tariffs, whereas export demands can profoundly affect economics and livelihood access, as has occurred in Uganda, Kenya, and various other regions (East African 2016, 2022, Monitor 2020). This is significant, because for economies such as Kenya, the export of cut flowers is now one of their greatest sources of income (following tourism and tea exports), and therefore, changes in demand patterns or poor production years can have severe socioeconomic impacts (Mohammed 2020).

The production of nonfood items for export can also remove key grazing lands from Indigenous peoples (Kavilu 2016). Areas now used for floriculture in Kenya were often previously used by the Maasai people (Kirigia et al. 2016), and groups were forced out of traditional lands and had access to water blocked because of floriculture, meaning they can no longer graze the cows they depend on (Food and Water Watch 2008, Kavilu 2016, Abate 2020). Furthermore, prior to conversion to floriculture, many of these areas either were used by smallholder farmers for local consumption or replaced natural habitat directly, and these processes are common for floriculture across the African continent (Abate 2020). Although new regulations have been set to address this, they have not yet been effective at reconciling the needs of local communities with the impacts of floriculture (Kavilu 2016). This has led to discussions on the conflict between flowers and food, with the most fertile lands being used for floriculture rather than to produce food for the local, national, or regional population. Furthermore, if the market fails, then there is neither food produced nor any way to access an income (Kirigia et al. 2016, Mohammed 2020).

Another key social issue is the lack of consideration of access and benefit sharing (ABS) in the ornamental plant industry. The importance of ABS has been acknowledged for some time, but the 2010 Nagoya Protocol on Access to Genetic Resources and the Fair and Equitable Sharing of Benefits Arising from their Utilisation put in place clear obligations for the sharing of both monetary and nonmonetary benefits from the use of a country's genetic resources (CBD 2011). However, there is still relatively little attention paid to this in high-value horticultural sectors such as orchids (Hinsley and Roberts 2018) and little data on how ABS will affect trade in the wider ornamental plant sector (Blackhall-Miles et al. 2023). The reliance on wild genetic material, especially in mass-produced cut flower and potted plant markets, is likely low, but ABS still applies in cases where novelty in species or color form is highly prized, which may lead to the use of wild plant resources (Laird and Wynberg 2012). The first ABS agreement in the ornamental plant sector, between the South African National Biodiversity Institute and a US horticultural company in 1999, highlighted the complexities of this process, particularly in ensuring adequate benefits to the source country of the plant material (CBD 2008). A lack of consideration of ABS in the sector has led to issues with alleged biopiracy and a loss of natural resources for low-income countries (Forestal 2023, Wynberg 2023).

Box 3. **Vertebrate hitchhikers detected in the Netherlands and the United Kingdom.**

**Figure 6.**
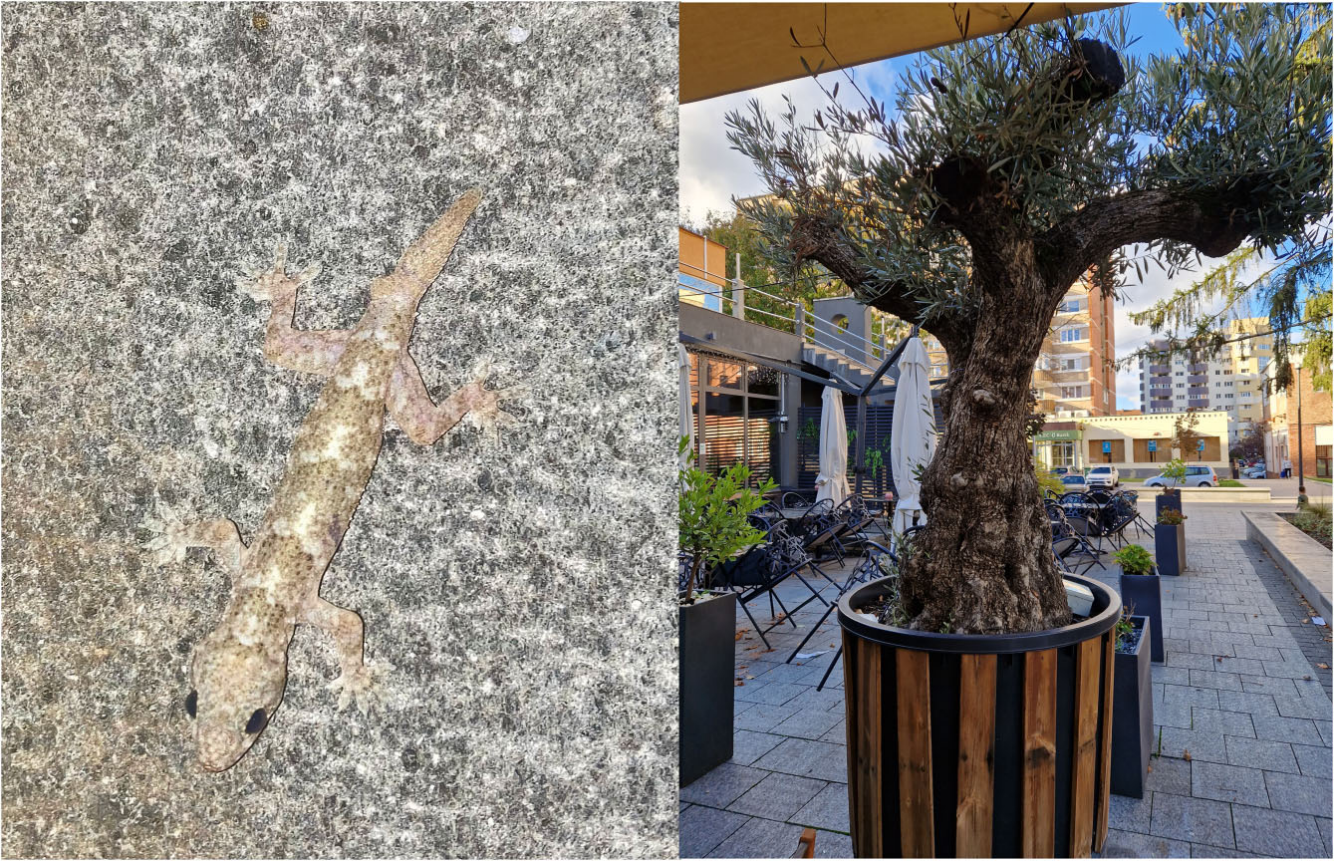
(a) Tropical house gecko (*Hemidactylus mabouia*) introduced in Madeira. (b) Ornamental olive trees imported in Romania. Credit Mihai Leu.

There are numerous accounts of live vertebrate hitchhikers (e.g., lizards, amphibians) accidentally moved between countries in fresh produce shipments including ornamental plants. Evidence now strongly indicates the live plant trade for ornamental purposes as one of the main forms of reptile introductions outside their range. In particular, trade of potted olive trees (figure 6b) from Italy and Spain appears responsible for the widespread introductions of the Italian wall lizard *Podarcis siculus*, Moorish gecko *Tarentola mauritanica*, and several species of continental European snakes (Silva-Rocha et al. 2015, Rato et al. 2023). However, such hitchhiker detections are often at the point of sale or following purchase and therefore remain unrecorded unless wildlife rehabilitation centers are contacted to collect the animals.

We analyzed two unpublished databases of exotic amphibians and reptiles accidentally discovered in the Netherlands and the United Kingdom, with observations submitted typically by members of the public and shopworkers, ornamental plant propagation facilities, and airports. The observations spanned 1995–2023 but most were after 2015, probably reflecting higher recent interest in recording such data. Of the 82 incidents reported, we used the descriptive text to manually identify and remove those referring to food or other nonornamental plant products. The remaining 56 incidents were all linked to ornamental plant trade, but, in addition, there were many of unclear origin, imported with “fresh produce” or simply reported by airport animal receptions. Outside of these data sets, in the United Kingdom in 2016–2017, there were additional live discoveries in florist shops and garden centers of *Hyperolius marmoratus*, a reed frog imported with aquatic ornamental plants from South Africa, a *Dendrosophus norandinus* endemic tree frog imported with roses from Colombia via Ecuador, and another *Hyperolious* species imported in heliconias from the Ivory Coast in 2017.

The interceptions from the Netherlands included six amphibian species, four imported with unspecified ornamental plants from Costa Rica (*Smilisca* sp., *Smilisca baudinii, Pristimantis* sp., and Centrolenidae), one European treefrog (*Hyla arborea*) found in a shipment of ornamental plants, but also two giant toads (*Rhinella horribilis*) from Costa Rica and others of unknown country origin, probably also imported with ornamental plants.

The main reptile species detected with ornamental plants was the Italian wall lizard (16 in the Netherlands and one in the United Kingdom, imported from the Netherlands, where the species does not naturally occur, so it was most likely reexported from there), but also two European wall lizards *Podarcis muralis*. Almost all of these animals were probably transported with ornamental trees, although this could not be verified with certainty, because they were found within wider shipments of ornamental plants. Four species, two lizards and two snakes, originated from southern Europe (one each of *Hemidactylus turcicus*, from Italy; *Psammodromus algirus* and *Malpolon monspessulanus*, from Portugal; and *Natrix maura*, exact country unknown). One slow worm (*Anguis fragilis*) moved in ornamental plants most likely originated from the Netherlands.

However, in the detections among ornamental plants in the Netherlands there were also several species from outside Europe including geckos (*Hemidactylus frenatus, n* = 1; *Hemidactylus platyurus, n* = 3; *Hemidactylus mabouia, n* = 1; *Gekko badenii, n* = 1; *Tarentola delalandii, n* = 1; *Thecadactylus rapicauda, n* = 1; *Gehyra mutilata, n* = 1; figure 6a), other lizards from the Western Hemisphere (three *Anolis sagrei* and one each of *Anolis equestris, Anolis biporcatus, Anolis carolinensis*, and *Lepidophyma flavimaculatum*), and the snakes *Sibon nebulatus* (*n* = 2)*, Oligodon octolineatus* (*n* = 1) and *Indotyphlops braminus* (*n* = 1).

In the UK data set, there were 25 other reptile records, mainly of geckos *Hemidactylus* spp. and *Tarentola mauritanica* but also several non-European amphibians (*Hyperolius* spp. and *Osteopilus* spp.), yet the exact means of introduction were unknown as they were reported at the airport animal reception center.

## Impacts on importers

The impacts on importing countries can vary widely, and need to be carefully considered to enable targetted management of risks.

### Biosecurity, invasive species and risk pathways

These impacts of the trade are better understood, with various ornamental plants known for becoming invasive (Padilla and Williams 2004, Dehnen-Schmutz et al. 2007), including many with severe financial and ecological consequences, such as *Fallopia japonica, Pueraria montana, Crassula helmsii*, and *Hydrocotyle ranunculoides* (Aguilera et al. 2010, Hussner 2012, Harron et al. 2020), or driving the introductions of plant diseases (Hammond et al. 2023) and pests (Patoka et al. 2016). For example, in a study assessing the link between horticulture and invasive species spread, 73 of the 89 plant species included were likely to have invaded as a result of horticulture (Beaury et al. 2023), and horticulture remains a main pathway of introduction for invasive plants (Van Kleunen et al. 2018). This is becoming increasingly relevant in relation to climate change, with ornamental plants traded from nurseries in the United States—for instance, representing a major risk of spreading invasive plants and pathogens under both current and future climates (Beaury et al. 2023). Nearly 20 years ago, a review of plant pathogen introductions in Great Britain in 1970–2004 concluded that, although 14% of the known introduction pathways related to imports of vegetative plants and therefore represented a significant proportion, nearly 80% of pathogen introduction pathways could not be exactly identified (Jones and Baker 2007). Because the production of ornamental plants has greatly expanded in recent decades, including in tropical regions because of trade globalization and the reduced costs of production (Wainwright et al. 2014), novel export chains have facilitated the transport of a new set of nonnative and sometimes invasive species across the globe (Hulme 2021). Such species arrive either directly as the traded species themselves or, more commonly, by riding on shipment means—for instance, via biofouling, within packing materials, or as contaminants of the products, such as insects or their eggs on traded fresh plant material (Zieritz et al. 2017).

The international trade of ornamental plants has long been linked to biosecurity trade risk pathways (Brasier 2008, Dodd et al. 2015, Hulme et al. 2018), with surveys showing a steady increase in both the volumes of ornamental plants imported in specific European countries and a high and increasing number of associated “contaminants,” typically insects but also other taxa, imported alongside the traded plants via their root system, the compost material, and the plant itself (Staverløkk and Sæthre 2007). For instance, dozens of species of carnivorous flatworms have been introduced in various parts of the world with potted plants and potting substrate, including, in recent years, in Europe, with potentially significant impacts on native biodiversity (Alvarez-Presas et al. 2014, Justine et al. 2018, Mori et al. 2022). Similarly, reports from various countries suggest that the horticultural industry is perhaps the most important vector for introductions of small snails and slugs. For example, 31 such terrestrial species, all but two of them alien and five previously unrecorded, were found in the nurseries of native Hawaiian plants destined for local restoration projects (Cowie et al. 2008). However, although the impact costs are relatively well established for agriculture, industry (e.g., water companies or forestry), and property, the financial costs in terms of biodiversity impacts from nonnative invasive species remain comparatively poorly understood and hard to calculate and are therefore often excluded (Hanley and Roberts 2019, Cuthbert et al. 2021). However, with better compilations on the costs of invasive species (e.g., Diagne et al. 2020), such assessments are becoming more feasible. There is also potential to facilitate introductions of human disease vectors into new areas via the transport of ornamental plants, as was shown for the introductions of *Aedes albopictus* mosquito in the Netherlands from Asia via imports of lucky bamboo plants (*Dracaena sanderiana*; Scholte et al. 2008), and, although they are rare, those costs could be substantial.

The global establishment of nonnative and invasive species is predicted to keep growing, despite the increasing focus on better managing risks and pathways from a policy perspective (Hulme 2009, Lodge et al. 2016). Many widespread invasive species (e.g., hemipterans such as the brown marmorated stink bug, *Halyomorpha halys*) are still globally expanding because of accidental introductions and bridgehead effects (i.e., where new invasions arise from particularly successful invasive populations that serve as the source in colonizing remote new territories, rather than from the original native range), with substantial costs to agriculture, human nuisance and also indirect biodiversity impacts because of the increased need for pesticide treatments (Leskey and Nielsen 2018). Other nonnative species have become established in new areas but still have relatively unknown environmental impacts, although they include potentially substantial changes to ecosystem functioning, such as invasive earthworms in the Arctic tundra (Blume-Werry et al. 2020). In several cases, new species were first described as nonnative, without definite knowledge of their native range, such as the carnivorous ghost slug *Selenochlamys ysbryda* incidentally detected in a garden in Wales, in the United Kingdom, but presumed to originate in the Caucasus and introduced as a contaminant of ornamental plants (Rowson and Symondson 2008). Many introduced nonnative species can also harbor pathogens or parasites that are harmless to the invasive host but lethal to indigenous species (see box 3; Vilcinskas 2015).

More recently, specific risks from hybridization have been highlighted for some invasive species, with potential for biodiversity loss but also for acquiring significant new traits. For example, the hybridization of the introduced invasive *Helicoverpa armigera* moths with the native agricultural pest *Helicoverpa zea* in Brazil conferred insecticide resistance and so hindered effective pest management (Valencia-Montoya et al. 2020). Many invasive species have also proven impossible to eradicate once they are established, requiring indefinite control, which is difficult to fund and maintain, especially when the impact is on biodiversity rather than on economic activities. This highlights the crucial role of prevention and robust identification of risk and adequate mitigation of these risks, including for the ornamental plant trade (Ahmed et al. 2022).

Furthermore, the introduction of zoonotic diseases via reptiles or amphibians in ornamental plants needs to be considered; the rat lung worm (*Angiostrongylus cantonensis*), a causative agent of eosinophilic meningitis in humans, has been found in the highly abundant lizard *Gallotia galloti* on Tenerife, where it functions as a paratenic host, with rats being the definitive host (Anettová et al. 2022). Introductions via either infected lizards or mollusks hitchhiking in ornamental plants from Tenerife and, more recently, also Mallorca should therefore be considered by medical practitioners and plant inspectors. Recently, this parasite has been found on mainland Europe, in Valencia, Spain (see box 1; Paredes-Esquivel et al. 2023).

## Future priorities to reduce risks

We have shown that the global ornamental plant and cut flower market has doubled in value over the last two decades and has demonstrated changing patterns of export and import, including increasing exports from Africa and South America and emerging Asian import markets. Furthermore, the global issues affecting the markets in recent years, such as the COVID-19 pandemic, the blocked shipping via the Suez Canal, and the war in Ukraine, have all highlighted the complexity of global trading patterns, their mutability, and their vulnerability to sudden shocks and novel risks (Christie et al. 2022, Hellegers 2022). There is a need to take a proactive approach to better understand the potential risks associated with changing supply chains and emerging markets, especially because differences in consumer demand coupled with supply factors are likely to mean that growing markets have different characteristics from established European markets (Yuan et al. 2021). For importers of ornamental plants, a trade that has complex specific risks and challenges, new markets should be carefully monitored to ensure that they develop sustainably, in a way in which biosecurity risks and environmental costs are minimized. However, diligent considerations of the impacts on exporter countries are also urgently needed, both to prevent the illegal or unsustainable harvest of native species (e.g., vulnerable species of orchids and succulents) and to reduce the wider environmental, food sustainability, and social impacts of producing large volumes of flowers for export.

The changing patterns of global flower trade that we highlight in this review have the potential to increase the risks to wild plants from trade, in both supplier and consumer nations. Changing markets may bring new species into trade, potentially leading to unsustainable levels of harvest being reached before legal and management protections for wild species can be put in place. The current regulations on the international trade via CITES include all species of some of the taxa most frequently cited as at threat from wild harvest for trade, including orchids, cacti, and cycads (CITES 2023). However, we have shown that, despite these taxa being listed on CITES for almost 50 years and illegal trade in wild plants clearly taking place on a large scale (Hinsley et al. 2017, Gale et al. 2019, Margulies et al. 2023), comparatively few seizures of ornamental plants are reported. Although we used only one seizures database, the seizure data are broadly known to be incomplete and influenced by enforcement effort and reporting biases (Challender et al. 2022, Donald et al. 2024). These shortfalls represent major barriers to understanding the extent and nature of the illegal and wild plant harvest: the lack of data on what is being traded. This, coupled with an absence of population data for which to assess sustainability of harvest for trade (Ticktin et al. 2023), is likely to leave many species of ornamental plants vulnerable to overharvest, even where strict regulations exist.

Shifting markets also present increased threats for biosecurity, because the ornamental plant trade now involves a much higher diversity of exporting countries, including increasing exports from tropical areas, larger volumes, and shorter times in transit. Our analyses of novel interception data sets show several major pest species for agriculture and forestry being detected in ornamental plant shipments in both the Netherlands and the United Kingdom in the last decade, as well as a variety of live amphibians and reptiles. The interceptions we have reported in the present article likely represent only a proportion of actual pests and hitchhiker species, given that the shipments are destroyed once notifiable species have been detected (DEFRA 2023) and that vertebrate detections are opportunistic and poorly recorded. The volumes of plants in trade are currently so large that contaminant interception with high certainty is logistically difficult (and likely inadequately resourced), and this will be exacerbated as the market for internationally traded plants grows. Some of the highest numbers of interceptions came from key products such as roses being exported from emerging growing countries, such as Kenya and Uganda, although such data is prone to a shifting focus in checks and other sources of potential bias.

Our analysis suggests that substantial opportunities remain for pests, including insects, pathogens, fungi, and gastropods, to be transported alive and to potentially establish populations in new areas, particularly for ornamental plants destined for planting either in greenhouses or outdoors, as well as some risk for various small vertebrate species introductions. Several amphibian and reptile species are rapidly expanding their ranges as introduced species, some apparently harmless but others with biodiversity impacts, especially *Podarcis siculus* and *Anolis sagrei*, which have become invasive in many regions, whereas herpetofauna species are directly relevant for the introduction of novel pathogens such as ranaviruses or fungal diseases (Norval et al. 2013, Kolby 2016, García‐Díaz et al. 2017, Stroud et al. 2017). Even after imported potted plants die, some risks persist if the contaminated plants are disposed of in compost heaps or organic waste bins. This is not a new issue; a review in Great Britain of data from 1970–2004 concluded that “the large volumes of ornamentals traded around the world cannot adequately be inspected [or] monitored and influxes of previously unknown pathogens (some of which can attack more important hosts) may occur on them from time to time,” whereas exotic pathogens could be introduced prior to understanding the risks they pose and before legislation targeting them can be introduced (Jones and Baker 2007). Although policy, strategy, and regulations have all improved in recent years, the risks are also increasing, and it is not possible to assess whether these are being mitigated.

Important changes to reduce risk pathways from ornamental plants include introducing plant passports or phytosanitary certificates and regulations restricting the types of potting substrate that can be used, because soil, compost, and other organic material has been shown to be a key source of contaminants in ornamental plant trade (Staverløkk and Sæthre 2007). However, the trade logistics mean that despite improvements in biosecurity protocols the number of plant contaminants remains high and the taxonomic uncertainty (numerous contaminants where identification was not possible) means that robust risk estimations are inherently difficult. Phytosanitary inspections are subject to internationally adopted standards developed by the FAO (Food and Agriculture Organization of the United Nations). The guidelines for administrative and physical checks are defined in ISPM 23 (International Standards for Phytosanitary Measures, FAO 2005) whereas the methodology of sampling of consignments is prescribed in ISPM 31 (FAO 2009). The risk aspects involved in growing media in association with plants for planting is addressed in ISPM 40 (FAO 2017b). In addition, it is important to remember that most notifiable pests are categorized as such for their impact on agriculture and forestry and much less information exists for impacts on wild plants. The main species found as contaminants were unsurprisingly cosmopolitan invasive pests, such as *Bemisia tabaci*, which are extremely polyphagous, necessitating increased attention to certain import markets (Royal Flora Holland 2023).

There are various practical problems in implementing robust policy for ornamental plant risks when dealing with biodiversity. For instance, many invasive flatworms, including the New Guinea flatworm *Platydemus manokwari*, are not agricultural pests and do not have a direct impact on plants, including ornamental plants, but they are an important threat to biodiversity as predators of snails (Justine et al. 2014). As a result, the responsibility for managing such invasive species may fall between the remits of agricultural and environmental regulatory bodies (Justine et al. 2014), hampering action and exacerbated by the lack of financial costs of biodiversity loss from invasive species. Similarly, the prevention of introduction and establishment is much more effective than nonnative species control, given that, although terrestrial plants, for instance, could be a high priority for eradication, there are few recorded eradications of these species in Britain or Europe, probably because they are overlooked at the early stages invasion, meaning that management decisions are taken too late for eradication to be feasible or cost effective (Booy et al. 2017).

Although we have identified a range of issues related to the ornamental plant trade, there are limitations that may influence our findings, including those associated with the paucity of research on certain threats and the availability of raw data on plant trade. For example, although case studies exist that describe the illegal plant trade in certain places (e.g., China, Gale et al. 2019), little is known about the global volume, species, and locations of international illegal trade. Furthermore, data on contaminant interceptions were not available for many countries that we approached for this analysis, partly because of government policies and sensitivities around sharing of these data, even when anonymized to exclude data on specific traders. Where interception data were available, the taxonomic identification of contaminants was not always at species level, or the data on the provenance was missing. Moreover, it was sometimes unclear whether the purpose of the shipment in which a contaminant was intercepted was ornamental, making it difficult to establish the relative role that ornamental trade played in the movement of contaminants. For example, olive trees, which are a potential host plant for the plant-disease-causing bacterium *Xylella fastidiosa*, may be traded for both agricultural and ornamental purposes, which were difficult to separate out in our analysis and which can be subject to different trading regulations and strategies. Finally, in countries such as the United Kingdom, where interception data are made publicly available, they are often restricted to notifiable species, with other contaminant interceptions not published or even recorded, despite the fact that the list of pests is dynamic.

Wider environmental challenges arise if flower growing displaces food production or diverts important or insufficient water resources in areas around the globe. The use of water for floriculture is already considerable, and with the changing climate and the growing potential for water scarcity, this could be very problematic in the future (Boretti and Rosa 2019). Furthermore, competition for land and access to water have led countries to prioritize the production of flowers for export over food for domestic consumption (Mena-Vásconez et al. 2016), undermining food security and rendering populations vulnerable to market shocks. The depletion of aquifers is a major and overlooked dimension of floriculture, which requires more consideration if we are to reduce the burden of production within producer nations (Manasi and Raju 2020, Bellwood-Howard et al. 2022). Furthermore, the often weak regulation on agrochemicals pollutes water courses and soil, as well as having major implications for ecosystem and species health in producer regions (Pereira et al. 2021). Finally, little attention has been paid to the potential of supplier nations to also be vulnerable to invasion and to naturalization of the nonnative plants being cultivated, and this also poses a particular risk in many tropical regions (Junqueira and Peetz 2018).

On the basis of current trends, it is likely that ornamental plant and flower production will continue to increase across much of Africa and South America and that their importers will grow and diversify, especially in regions such as Asia. Although some elements of unsustainability, such as water use or carbon footprint because of long distance air transportation of cut flowers, are harder to remedy, more sustainable modes of production which minimize impacts on supplier nations are possible. Mitigating impacts remains a major challenge for policy-making, although recommendations on life-cycle assessment (LCA), integrated pest management, and private voluntary standards on the incorporation of environmental standards have been issued, such as the Floriculture Sustainability Initiative standards noted in the GlobalGAP report (Wainwright et al. 2014; https://globalgapsolutions.org). Standards and certification, such as the floriculture sustainability certification scheme of the GlobalGAP report, provide a significant step forward in reducing the impacts of floriculture on supplier countries, but as yet, only a small fraction of floriculture farms have been certified through this approach (https://floriculture.ggn.org/Flori/Index?lang=en). These standards include food safety, traceability, worker welfare and safety, and environmental and resource protection, with over 158 principles to ensure that farms are genuinely sustainable (GlobalGAP 2022). They could become a basic core standard for importer nations from suppliers to begin to institute serious measures to reduce the impacts of floriculture trade. More recently, a new global standard is being developed, FloriPEFCR (Cut Flowers and Potted Plants Product Environmental Footprint), which is a method based on LCA to provide a single certification standard for the sustainability of imported floricultural products (Wageningen University and Research 2023). Previous LCAs for cut flowers have been focused primarily on carbon footprint (Lan et al. 2022), and new methods should be focused on developing a more holistic gauge of impacts. Furthermore, there have been wider efforts for the sustainable harvest of medicinal and aromatic plants, such as the FairWild Standard, which can incentivize legal and sustainable over illegal harvest (Morgan and Timoshyna 2016, Timoshyna and Drinkwater 2021), and lessons from these should be applied to horticultural plants, in cases where sustainable wild harvest is possible.

## Conclusions

Our analyses on the myriad risks from the ornamental plant trade suggest that the lack of standardized data collection and sharing on these issues could represent a significant issue for quantifying risk pathways, and efforts should be made to make available and collate these data sets to adequately inform policy. Although the ornamental plant industry brings many benefits, its potential and recorded impacts on wild species, ecosystems, and people in both exporting and importing nations means that careful management of these growing markets is essential.

Newer standards may rebalance some of these negative impacts, but the recognition of the multifaceted challenges are needed to develop appropriate solutions, especially overlooked issues such as food security, Indigenous rights, and local biodiversity loss. Although higher standards could reduce pesticide imports (especially for banned pesticides and other chemicals), efforts to reduce pests often fall largely on supplier nations (Chege et al. 2021), although importer nations may also increase the rate of checks when consistent problems are identified (Royal Flora Holland 2023). Inconsistency in reporting, especially with regards to notifiable or regulated pests whose definition varies over time, means that distinguishing true trends from recorded information is difficult. Given the number and diversity of vertebrates, including fragile ones such as tropical frogs, reported live in imported products, the number of imported invertebrate pests is likely underestimated, and more consistent measures are needed to provide an accurate understanding of the true implications of trade and how they might be managed.

## Supplementary Material

biae124_Supplemental_File

## Data Availability

All openly available datasets analysed in this project have been linked to in the text. The Netherlands interception data are not publicly available, but can be obtained on request from RAVON (https://www.ravon.nl) and the Netherlands Institute for Vectors, Invasive plants and Plant health (NIVIP) (https://english.nvwa.nl/topics/nivip). The UK vertebrate interception data can be requested from the National Centre for Reptile Welfare at https://www.ncrw.co.uk/.

## Author Biography

Amy Hinsley (amy.hinsley@biology.ox.ac.uk) is affiliated with the Department of Biology at the University of Oxford, in Oxford, England, in the United Kingdom. Alice Hughes (achughes@hku.hk) is affiliated with School of Biological Sciences at the University of Hong Kong, in Hong Kong, the People's Republic of China. Johan van Valkenburg (j.l.c.h.vanvalkenburg@nvwa.nl) is affiliated with the Netherlands Institute for Vectors, Invasive Plants, and Plant Health, in Wageningen, in the Netherlands. Tariq Stark (t.stark@ravon.nl) is affiliated with Reptile, Amphibian, and Fish Conservation Netherlands, in Nijmegen, in the Netherlands. Jeroen van Delft (j.v.delft@ravon.nl) is affiliated with the Reptile, Amphibian, and Fish Conservation Netherlands and with the Netherlands Centre of Expertise on Exotic Species, in Nijmegen, in the Netherlands. William Sutherland (wjs32@cam.ac.uk) and Silviu Petrovan (sop21@cam.ac.uk) are both affiliated with the Department of Zoology and with BioRISC, at the University of Cambridge, in Cambridge, England, in the United Kingdom.

## References

[bib1] Abate AG. 2020. The effects of land grabs on peasant households: The case of the floriculture sector in Oromia, Ethiopia. African Affairs 119: 90–114.

[bib2] Adamopoulou C , Pafilis P. 2019. Eaten or beaten? Severe population decline of the invasive lizard *Podarcis siculus* (Rafinesque-Schmaltz, 1810) after an eradication project in Athens, Greece. Herpetozoa 32: 165–169.

[bib3] Aguilera AG , Alpert P, Dukes JS, Harrington R. 2010. Impacts of the invasive plant *Fallopia japonica* (Houtt.) on plant communities and ecosystem processes. Biological Invasions 12: 1243–1252.

[bib5] Ahmadalipour A , Moradkhani H. 2018. Multi-dimensional assessment of drought vulnerability in Africa: 1960–2100. Science of the Total Environment 644: 520–535. 29990902 10.1016/j.scitotenv.2018.07.023

[bib4] Ahmadalipour A , Moradkhani H, Castelletti A, Magliocca N. 2019. Future drought risk in Africa: Integrating vulnerability, climate change, and population growth. Science of the Total Environment 662: 672–686. 30703725 10.1016/j.scitotenv.2019.01.278

[bib6] Ahmed DA. et al. 2022. Managing biological invasions: The cost of inaction. Biological Invasions 24: 1927–1946. 10.1007/s10530-022-02755-0.

[bib7] Alexander PD , Bragg NC, Meade R, Padelopoulos G, Watts O. 2008. Peat in horticulture and conservation: The UK response to a changing world. Mires and Peat 3: 8.

[bib8] Alvarez-Presas M , Mateos E, Tudo A, Jones H, Riutort M. 2014. Diversity of introduced terrestrial flatworms in the Iberian Peninsula: A cautionary tale. PeerJ 2: e430. 24949245 10.7717/peerj.430 PMC4060057

[bib9] Anettova L , Izquierdo-Rodriguez E, Foronda P, Baláž V, Novotný L, Modrý D. 2022. Endemic lizard *Gallotia galloti* is a paratenic host of invasive *angiostrongylus cantonensis* in Tenerife, Spain. Parasitology 149: 934–939 10.1017/S0031182022000336 PMC10090600 35321776

[bib38] Australian Department of Agriculture . 2019. Final Pest Risk Analysis for Cut Flower and Foliage Imports—Part 1. Commonwealth of Australia.

[bib11] Averyanov LV , Pham VT, Ke LP, Tien HN, Xuan CC, Tien VN, Quan HN. 2014. Field survey of *Paphiopedilum canhii*: From discovery to extinction. Slipper Orchids 24: 16–26.

[bib12] Bär J et al. 2022. Pesticide Atlas: Facts and Figures about toxic Chemicals in Agriculture, 2nd ed. 2022. Heinrich-Böll-Stiftung, Friends of the Earth Europe, Bund für Umwelt und Naturschutz, PAN Europe https://eu.boell.org/sites/default/files/2023-04/pesticideatlas2022_ii_web_20230331.pdf.

[bib13] Barriga M. 2006. El Polen se Va y No Vuelve: La Deuda Ecológica de la Floricultura Colombiana. Censat Agua Viva. https://cdn.biodiversidadla.org/content/download/23420/106775/file/La+deuda+ecol%C3%B3gica+de+la+floricultura+colombiana.pdf.

[bib15] Beaury EM , Patrick M, Bradley BA. 2021. Invaders for sale: The ongoing spread of invasive species by the plant trade industry. Frontiers in Ecology and the Environment 19: 550–556.

[bib14] Beaury EM , Allen JM, Evans AE, Fertakos ME, Pfadenhauer WG, Bradley BA. 2023. Horticulture could facilitate invasive plant range infilling and range expansion with climate change. BioScience 73: 635–642.

[bib16] Bek D , Binns T, Blokker T, McEwan C, Hughes A. 2017. A high road to sustainability? Wildflower harvesting, ethical trade and social upgrading in South Africa's Western Cape. Journal of Agrarian Change 17: 459–479.

[bib17] Bellvert A , Arnedo MA. 2016. Threatened or threatening? Evidence for independent introductions of *Macrothele calpeiana* (Walckenaer, 1805; Araneae: Hexathelidae) and first observation of reproduction outside its natural distribution range. Arachnology 17: 137–141.

[bib18] Bellwood-Howard I et al. 2022. A multicriteria analysis of groundwater development pathways in three river basins in sub-Saharan Africa. Environmental Science and Policy 138: 26–43.

[bib17a] Blackhall-Miles RJ , Fraser IM, Roberts DL. 2023. Investigating consumer preferences for known wild provenance of plants within the horticultural trade. Plants, People, Planet 5: 398–407.

[bib19] Berners-Lee M. 2020. How Bad Are Bananas? Profile Books.

[bib20] Blume-Werry G , Krab EJ, Olofsson J, Sundqvist MK, Väisänen M, Klaminder J. 2020. Invasive earthworms unlock arctic plant nitrogen limitation. Nature Communications 11: 1766. 10.1038/s41467-020-15568-3 PMC7156384 32286301

[bib21] Booy O et al. 2017. Risk management to prioritise the eradication of new and emerging invasive non-native species. Biological Invasions 19: 2401–2417.

[bib22] Boretti A , Rosa L. 2019. Reassessing the projections of the world water development report. NPJ Clean Water 2: 15.

[bib23] Bouwman H et al. 2019. Evidence of impacts from DDT in pelican, cormorant, stork, and egret eggs from KwaZulu-Natal, South Africa. Chemosphere 225: 647–658. 30901658 10.1016/j.chemosphere.2019.03.043

[bib25] Brasier CM. 2008. The biosecurity threat to the UK and global environment from international trade in plants. Plant Pathology 57: 792–808.

[bib26] Button K. 2020. The economics of Africa's floriculture air-cargo supply chain. Journal of Transport Geography 86: 102789. 32834672 10.1016/j.jtrangeo.2020.102789 PMC7336125

[bib28] [CBD] Convention on Biological Diversity. 2008. Ball Horticulture and the South African National Biodiversity Institute. CBD. www.cbd.int/doc/meetings/abs/abswg-06/other/abswg-06-cs-04-en.pdf.

[bib150] [CBD] Convention on Biological Diversity. 2011. The Nagoya Protocol on Access and Benefit-Sharing. CBD. www.cbd.int/abs/infokit/revised/web/factsheet-nagoya-en.pdf.

[bib29] Challender DW et al. 2022. Mischaracterizing wildlife trade and its impacts may mislead policy processes. Conservation Letters 15: e12832.

[bib30] Chege F , Bundi M, Kisingiri JB, Nekambi E. 2021. Assessing the Impact of Strengthening the Phytosanitary Capacity of the Floriculture Sector in Uganda. CABI. Working Paper 17. 10.1079/CABICOMM-62-8143.

[bib31] Christie AP , Aldridge DC, Gallardo B, Ó hÉigeartaigh S, Petrovan SO, Sutherland WJ. 2022. Strengthen biosecurity when rewiring global food supply chains. Nature 606: 864–864. 10.1038/d41586-022-01773-1. 35764802

[bib32] [CITES] Convention on International Trade in Endangered Species of Wild Fauna and Flora. 2023. Appendices I, II, and III. CITIES. https://cites.org/eng/app/appendices.php. 10.1159/000459796 712806

[bib33] Cowie RH , Hayes KA, Tran CT, Meyer WM. III 2008. The horticultural industry as a vector of alien snails and slugs: Widespread invasions in Hawaii. International Journal of Pest Management 54: 267–276.

[bib34] Cuthbert RN et al. 2021. Global economic costs of aquatic invasive alien species. Science of the Total Environment 775: 145238. 33715860 10.1016/j.scitotenv.2021.145238

[bib35] Darras A. 2020. Overview of the dynamic role of specialty cut flowers in the international cut flower market. Horticulturae 7: 51.

[bib36] Darras AI. 2020a. Implementation of sustainable practices to ornamental plant cultivation worldwide: A critical review. Agronomy 10: 1570.

[bib36a] [DEFRA] Department for Environment Food and Rural Affairs . 2023. Alert List. https://planthealthportal.defra.gov.uk/trade/imports/alert-list/

[bib37] Dehnen-Schmutz K , Touza J, Perrings C, Williamson M. 2007. The horticultural trade and ornamental plant invasions in Britain. Conservation Biology 21: 224–231. 17298528 10.1111/j.1523-1739.2006.00538.x

[bib39] Diagne C et al. 2020. InvaCost, a public database of the economic costs of biological invasions worldwide. Scientific Data 7: 277. 32901023 10.1038/s41597-020-00586-z PMC7479195

[bib40] Dodd AJ , Burgman MA, McCarthy MA, Ainsworth N. 2015. The changing patterns of plant naturalization in Australia. Diversity and Distributions 21: 1038–1050.

[bib41] Dolan C , Humphrey J. 2000. Governance and trade in fresh vegetables: The impact of UK supermarkets on the African horticulture industry. Journal of Development Studies 37: 147–176.

[bib42] Donald PF et al. 2024. Assessing the global prevalence of wild birds in trade. Conservation Biology 38: e14350. 39248745 10.1111/cobi.14350

[bib43] Donohoe M. 2008. Flowers, diamonds, and gold: The destructive public health, human rights, and environmental consequences of symbols of love. Human Rights Quarterly 30: 164.

[bib161] East African . 2016. Uganda flower farmers facing tough times as exports and earnings drop. East African (20 February 2016).

[bib162] East African . 2022. Kenya and Uganda cry foul as reality of new taxes checks in. East African (10 July 2022). https://www.theeastafrican.co.ke/tea/business-tech/kenya-and-uganda-cry-foul-as-reality-of-new-taxes-checks-in-3873944.

[bib45] [FAO] Food and Agriculture Organziation of the United Nations . 2005. Guidelines for Inspection. FAO. International Standard for Phytosanitary Measures no. 23. www.fao.org/3/j5062e/j5062e.pdf.

[bib46] [FAO] Food and Agriculture Organziation of the United Nations . 2009. Methodologies for Sampling of Consignments. FAO. International Standard for Phytosanitary Measures no. 31. www.fao.org/3/j5062e/j5062e.pdf.

[bib47] [FAO] Food and Agriculture Organziation of the United Nations . 2011. The State of the World's Land and Water Resources for Food and Agriculture: Managing Systems at Risk. FAO. www.fao.org/3/i1688e/i1688e.pdf.

[bib48] [FAO] Food and Agriculture Organziation of the United Nations . 2017a. Water for Sustainable Food and Agriculture: A Report Produced for the G20 Presidency of Germany. FAO. https://openknowledge.fao.org/server/api/core/bitstreams/b48cb758-48bc-4dc5-a508-e5a0d61fb365/content.

[bib49] [FAO] Food and Agriculture Organziation of the United Nations . 2017b. International Movement of Growing Media in Association with Plants for Planting. FAO. International Standard for Phytosanitary Measures no. 40. www.fao.org/3/cb2620en/cb2620en.pdf.

[bib50] [FAO] Food and Agriculture Organziation of the United Nations . 2021. The State of the World's Land and Water Resources for Food and Agriculture: Systems at Breaking Point. 10.4060/cb7654en.

[bib51] [FFI] Fauna and Flora International . 2018. Sustainable Harvesting of Fynbos in South Africa's Cape Floral Kingdom. FFI. www.fauna-flora.org/wp-content/uploads/2023/05/Flower-Valley-Case-Study_July-2018.pdf.

[bib52] Food and Water Watch . 2008. Lake Naivasha Withering under the Assault of International Flower Vendors. Food and Water Watch, The Council of Canadians. www.foodandwatereurope.org/wp-content/uploads/2009/11/FoodandWaterEuropeLakeNavaisha.pdf.

[bib53] Forestal RL. 2023. Using automated patent landscaping and legal geography analysis to spot biopiracy activities in the island of Hispaniola. World Patent Information 72: 102174.

[bib54] Gale SW , Kumar P, Hinsley A, Cheuk ML, Gao J, Liu H, Liu ZL, Williams SJ. 2019. Quantifying the trade in wild-collected ornamental orchids in South China: Diversity, volume, and value gradients underscore the primacy of supply. Biological Conservation 238: 108204.

[bib55] García-Díaz P , Ross JV, Woolnough AP, Cassey P. 2017. Managing the risk of wildlife disease introduction: Pathway-level biosecurity for preventing the introduction of alien ranaviruses. Journal of Applied Ecology 54: 234–241.

[bib56] Gelaye Y. 2023. The status and natural impact of floriculture production in Ethiopia: A systematic review. Environmental Science and Pollution Research 30: 9066–9081. 36437364 10.1007/s11356-022-24279-9

[bib57] GlobalGAP . 2022. Integrated Farm Assurance Flowers and Ornamentals. GlobalGAP. Integrated Farm Assurance v6. www.globalgap.org/export/sites/default/.content/.galleries/Documents_Other/220512_IFA-FO-v6_presentation-full_EN.pdf.

[bib58] Goettsch B et al. 2015. High proportion of cactus species threatened with extinction. Nature Plants 1: 1–7. 10.1038/nplants.2015.142 27251394

[bib59] González-Andrade F , López-Pulles R, Estévez E. 2010. Acute pesticide poisoning in Ecuador: A short epidemiological report. Journal of Public Health 18: 437–442.

[bib60] Gooijer YM et al. 2019. Research on Exposure of Residents to Pesticides in the Netherlands: OBO Flower Bulbs [Onderzoek Bestrijdingsmiddelen en Omwonenden]. Utrecht University.

[bib61] Grandjean P , Harari R, Barr DB, Debes F. 2006. Pesticide exposure and stunting as independent predictors of neurobehavioral deficits in Ecuadorian school children. Pediatrics 117: e546–e556. 16510633 10.1542/peds.2005-1781

[bib62] Guaita-Pradas I , Rodríguez-Mañay LO, Marques-Perez I. 2023. Competitiveness of Ecuador's flower industry in the global market in the period 2016–2020. Sustainability 15: 5821.

[bib63] Gudeta DT. 2012. Socio-economic and Environmental Impact of Floriculture Industry in Ethiopia. Wageningen University and Research .

[bib64] Gutema A. 2012. The Expansion of Floriculture Industry and its Livelihood Impacts on Local People: The Case of Holeta Town and its Surrounding Areas, Oromia Regional State. Master's thesis, Addis Ababa University, Addis Ababa, Ethiopia. http://etd.aau.edu.et/handle/123456789/11446

[bib66] Haile GG et al. 2020. Projected impacts of climate change on drought patterns over East Africa. Earth's Future 8: e2020EF001502.

[bib67] Hammond J , Huang Q, Jordan R, Meekes E, Fox A, Vazquez-Iglesias I, Vaira AM, Copetta A, Delmiglio C. 2023. International trade and local effects of viral and bacterial diseases in ornamental plants. Annual Review of Phytopathology 61: 73–95. 10.1146/annurev-phyto-021621-114618 37257057

[bib68] Hanley N , Roberts M. 2019. The economic benefits of invasive species management. People and Nature 1: 124–137.

[bib69] Harfoot M , Glaser SA, Tittensor DP, Britten GL, McLardy C, Malsch K, Burgess ND. 2018. Unveiling the patterns and trends in 40 years of global trade in CITES-listed wildlife. Biological Conservation 223: 47–57.

[bib70] Harron P , Joshi O, Edgar CB, Paudel S, Adhikari A. 2020. Predicting kudzu (*Pueraria montana*) spread and its economic impacts in timber industry: A case study from Oklahoma. PLOS ONE 15: e0229835. 32176706 10.1371/journal.pone.0229835 PMC7075552

[bib71] Hatch N , Wells L. 2012. Multilevel environmental governance: The case of Ethiopian floriculture. Environmental policy update. Pages 31–64 in Adams P et al., eds. Environmental Policy Update 2012: Development Strategies and Environmental Policy in East Africa. Colby College Environmental Policy Group. https://web.colby.edu/eastafricaupdate2012/files/2011/12/CH2_Multilevel-Environmental-Governance_The-Case-of-Ethiopian-Floriculture1.pdf.

[bib72] Hellegers P. 2022. Food security vulnerability due to trade dependencies on Russia and Ukraine. Food Security 14: 1503–1510. 10.1007/s12571-022-01306-8. 35891962 PMC9304541

[bib73] Hinsley A , Lee TE, Harrison JR, Roberts DL. 2016. Estimating the extent and structure of trade in horticultural orchids via social media. Conservation Biology 30: 1038–1047. 26991837 10.1111/cobi.12721

[bib73a] Hinsley A , Roberts DL. 2018. The wild origin dilemma. Biological Conservation 217: 203–206.

[bib75] Hinsley A et al. 2017. A review of the trade in orchids and its implications for conservation. Botanical Journal of the Linnean Society 186: 435–455.

[bib74] Hinsley A , Willis J, Dent AR, Oyanedel R, Kubo T, Challender DW. 2023. Trading species to extinction: Evidence of extinction linked to the wildlife trade. Cambridge Prisms: Extinction 1: e10. 40078683 10.1017/ext.2023.7 PMC11895731

[bib76] Hughes A. 2001. Global commodity networks, ethical trade and governmentality: Organizing business responsibility in the Kenyan cut flower industry. Transactions of the Institute of British Geographers 26: 390–406.

[bib77] Hughes AC. 2023. The Post-2020 Global Biodiversity Framework: How did we get here, and where do we go next? Integrative Conservation 2: 1–9.

[bib78] Hulme PE. 2009. Trade, transport, and trouble: Managing invasive species pathways in an era of globalization. Journal of Applied Ecology 46: 10–18.

[bib80] Hulme PE. 2021. Unwelcome exchange: International trade as a direct and indirect driver of biological invasions worldwide. One Earth 4: 666–679.

[bib79] Hulme PE et al. 2018. Integrating invasive species policies across ornamental horticulture supply chains to prevent plant invasions. Journal of Applied Ecology 55: 92–98.

[bib81] Hussner A. 2012. Alien aquatic plant species in European countries. Weed Research 52: 297–306.

[bib82] International Trade Centre . 2024. Trade map: Trade statistics for international business development. International Trade Centre. www.trademap.org.

[bib83] Jiao M , Wang Y, Li T, Li R, Liu B. 2022. Riverine microplastics derived from mulch film in Hainan Island: Occurrence, source, and fate. Environmental Pollution 312: 120093. 36064060 10.1016/j.envpol.2022.120093

[bib83a] Jones DR , Baker RHA. 2007. Introductions of non-native plant pathogens into Great Britain, 1970-2004. Plant Pathology 56: 891–910.

[bib84] Joosten H. 2015. Peatlands, Climate Change Mitigation, and Biodiversity Conservation: An Issue Brief on the Importance of Peatlands for Carbon and Biodiversity Conservation and the Role of Drained Peatlands as Greenhouse Gas Emission Hotspots. Nordic Council of Ministers.

[bib85] Junqueira AH , Peetz MDS. 2018. Sustainability in Brazilian floriculture: Introductory notes to a systemic approach. Ornamental Horticulture 24: 155–162.

[bib86] Justine JL , Winsor L, Gey D, Gros P, Thévenot J. 2014. The invasive New Guinea flatworm Platydemus manokwari in France, the first record for Europe: Time for action is now. PeerJ 2: e297. 24688873 10.7717/peerj.297 PMC3961122

[bib87] Justine JL , Winsor L, Gey D, Gros P, Thévenot J. 2018. Giant worms chez moi! Hammerhead flatworms (*Platyhelminthes, Geoplanidae, Bipalium* spp., *Diversibipalium* spp.) in metropolitan France and overseas French territories. PeerJ 6: e4672. 29844951 10.7717/peerj.4672 PMC5969052

[bib88] Kameri-Mbote P , Odhiambo E. 2015. Not so rosy: Farm workers’ human right to water in the Lake Naivasha Basin. Pages 118–146 in Hellum A et al., eds. Water Is Life: Women's Human Rights in National and Local Water Governance in Southern and Eastern Africa. Weaver Press.

[bib89] Kavilu S. 2016. Kenya's flourishing flower sector is not all roses for Maasai herdsmen. Reuters (30 June 2016). www.reuters.com/article/us-kenya-landrights-idUSKCN0ZG0Z0.

[bib90] Kerkhoven PM , Hagman H, Elings A, Hamel E. 2014. Floriculture for the Republic of Rwanda. Tierra BV, Wageningen University and Research Greenhouse Horticulture, SHER Ingénieurs-Conseil s.a. https://edepot.wur.nl/326636.

[bib91] Kirigia E , Betsema G, Van Westen G, Zoomers A. 2016. Flowers for Food? Scoping Study on Dutch Flower Farms, Land Governance and Local Food Security in Eastern Africa. Land Governance for Equitable and Sustainable Development. www.landgovernance.org/wp-content/uploads/2019/09/20160210-LANDac_Flower-Report-WEB.pdf/

[bib92] Kolby JE. 2016. Pathways of Amphibian Chytrid Fungus Dispersal: Global Biosecurity and Conservation Implications. PhD dissertation, James Cook University, Townsville, Queensland, Australia.

[bib93] Krishnan A. 2018. The origin and expansion of regional value chains: The case of Kenyan horticulture. Global Networks 18: 238–263.

[bib94] Laird SA , Wynberg R. 2012. Diversity and change in the commercial use of genetic resources: Implications for access and benefit sharing policy. International Journal of Ecological Economics and Statistics 26: 2–15.

[bib95] Lan YC , Tam VW, Xing W, Datt R, Chan Z. 2022. Life cycle environmental impacts of cut flowers: A review. Journal of Cleaner Production 369: 133415.

[bib96] Lansink A , Bezlepkin I. 2003. The effect of heating technologies on CO_2_ and energy efficiency of Dutch greenhouse firms. Journal of Environmental Management 68: 73–82. 12767863 10.1016/s0301-4797(02)00233-5

[bib98] Leipold B , Morgante F. 2013. The impact of the flower industry on Kenya's sustainable development. International Public Policy Review 7: 1–31.

[bib99] Lenzen M , Moran D, Kanemoto K, Foran B, Lobefaro L, Geschke A. 2012. International trade drives biodiversity threats in developing nations. Nature 486: 109–112. 22678290 10.1038/nature11145

[bib100] Leskey TC , Nielsen AL. 2018. Impact of the invasive brown marmorated stink bug in North America and Europe: History, biology, ecology, and management. Annual Review of Entomology 63: 599–618. 10.1146/annurev-ento-020117-043226 29068708

[bib101] Liu H , Gale SW, Cheuk ML, Fischer GA. 2019. Conservation impacts of commercial cultivation of endangered and overharvested plants. Conservation Biology 33: 288–299. 30168202 10.1111/cobi.13216

[bib102a] Lodge DM et al. 2016. Risk analysis and bioeconomics of invasive species to inform policy and management. Annual Review of Environment and Resources 41: 453–488. PMC7326237 32607083

[bib102] Lottering S , Mafongoya P, Lottering R. 2021. Drought and its impacts on small-scale farmers in sub-Saharan Africa: A review. South African Geographical Journal 103: 319–341.

[bib103] Lu JL. 2005. Risk factors to pesticide exposure and associated health symptoms among cut-flower farmers. International Journal of Environmental Health Research 15: 161–170. 16134479 10.1080/09603120500105638

[bib105] Manasi S , Raju KV. 2020. Coping Mechanisms for Climate Change in Peri-Urban Areas. Springer.

[bib107] Margulies JD et al. 2019. Illegal wildlife trade and the persistence of “plant blindness.” Plants, People, Planet 1: 173–182.

[bib106] Margulies JD , Moorman FR, Goettsch B, Axmacher JC, Hinsley A. 2023. Prevalence and perspectives of illegal trade in cacti and succulent plants in the collector community. Conservation Biology 37: e14030. 36317724 10.1111/cobi.14030

[bib108] Marigi SN. 2017. Climate change vulnerability and impacts analysis in Kenya. American Journal of Climate Change 6: 52.

[bib109] Maunder M , Cowan RS, Stranc P, Fay MF. 2001. The genetic status and conservation management of two cultivated bulb species extinct in the wild: *Tecophilaea cyanocrocus* (Chile) and *tulipa sprengeri* (Turkey). Conservation Genetics 2: 193–201.

[bib110] Mazzucato M , Okonjo-Iewala N, Rockström J, Shanmugaratnam T. 2023. Turning the Tide: A Call to Collective Action. Global Commission on the Economics of Water. https://watercommission.org/wp-content/uploads/2023/03/Turning-the-Tide-Report-Web.pdf.

[bib111] Mekonnen MM , Hoekstra AY. 2014. Water conservation through trade: The case of Kenya. Water International 39: 451–468.

[bib112] Mena-Vásconez P , Boelens R, Vos J. 2016. Food or flowers? Contested transformations of community food security and water use priorities under new legal and market regimes in Ecuador's highlands. Journal of Rural Studies 44: 227–238.

[bib113] Mengistie BT. 2016. Environmental Governance of Pesticides in Ethiopian Vegetable and Cut Flower Production. PhD dissertation, Wageningen University and Research, Wangeningen, Gelderland, Netherlands. https://edepot.wur.nl/391632.

[bib114] Merga LB , Mengistie AA, Alemu MT, Van den Brink PJ. 2021. Biological and chemical monitoring of the ecological risks of pesticides in Lake Ziway, Ethiopia. Chemosphere 266: 129214. 33310518 10.1016/j.chemosphere.2020.129214

[bib115] Mohammed O. 2020. Kenya's flower exports wither as demand drops amid coronavirus pandemic. Reuters (20 March 2020). www.reuters.com/article/health-coronavirus-kenya-flowers-idUSL8N2BA7M8.

[bib116] Monitor . 2020. Flower exports drop by 90%, prices dip by half. Monitor (26 March 2020). www.monitor.co.ug/uganda/business/commodities/flower-exports-drop-by-90-prices-dip-by-half-1882560.

[bib117] Morgan B , Timoshyna A. 2016. Creating synergies between voluntary certification standards (VCS) and regulatory frameworks: Case studies from the FairWild Standard. Policy Matters 21: 111–125.

[bib118] Mori E et al. 2022. Discovering the Pandora's box: The invasion of alien flatworms in Italy. Biological Invasions 24: 205–216.

[bib119] Mrema EJ , Ngowi AV, Kishinhi SS, Mamuya SH. 2017. Pesticide exposure and health problems among female horticulture workers in Tanzania. Environmental Health Insights 11: 1178630217715237. 28690397 10.1177/1178630217715237 PMC5484550

[bib120] Munnia A , Puntoni R, Merlo F, Parodi S, Peluso M. 1999. Exposure to agrochemicals and DNA adducts in Western Liguria, Italy. Environmental and Molecular Mutagenesis 34: 52–56. 10462724 10.1002/(sici)1098-2280(1999)34:1<52::aid-em8>3.0.co;2-a

[bib121] Negatu B , Dugassa S, Mekonnen Y. 2021. Environmental and health risks of pesticide use in Ethiopia. Journal of Health Pollution 11: 210601. 34267988 10.5696/2156-9614-11.30.210601 PMC8276724

[bib122] Netherlands Food and Consumer Product Safety Authority . 2020. Advisory Report by BuRO on the Risks of the Ornamental Horticulture Production Chain. Office for Risk Assessment and Research. https://english.nvwa.nl/documents/plant/plant-health/pest-risk-analysis/documents/advisory-report-by-buro-on-the-risks-of-the-ornamental-horticulture-production-chain.

[bib123] Newman C. 2002. Gender, time use, and change: The impact of the cut flower industry in Ecuador. World Bank Economic Review 16: 375–395.

[bib124] Norval G , Ivanova LV, Mao JJ. 2013. Greenhouses as potential reservoirs for the brown anole (*Anolis sagrei* Duméril and Bibron, 1837), an exotic invasive lizard in southwestern Taiwan. Reptiles and Amphibians 20: 199–202.

[bib125] [OEC] Observatory of Economic Complexity . 2023a. Which countries export live trees, plants, bulbs, cut flowers, and ornamental floliage? (2021). OEC. https://oec.world/en/visualize/tree_map/hs92/export/show/all/206/2021.

[bib126] [OEC] Observatory of Economic Complexity . 2023b. Which countries export live trees, plants, bulbs, cut flowers, and ornamental floliage? (2012–2021). OEC. https://oec.world/en/visualize/stacked/hs92/export/show/all/206/2013.2021.

[bib127] Özcerkes M. 2018. Kenya's Flower Industry Threatens Country's Fisheries. International Climate Initiative. www.international-climate-initiative.com/en/iki-media/video/kenyas_flower_industry_threatens_countrys_fisheries.

[bib128] Padilla DK , Williams SL. 2004. Beyond ballast water: Aquarium and ornamental trades as sources of invasive species in aquatic ecosystems. Frontiers in Ecology and the Environment 2: 131–138.

[bib129] Paini DR , Sheppard AW, Cook DC, De Barro PJ, Worner SP, Thomas MB. 2016. Global threat to agriculture from invasive species. Proceedings of the National Academy of Sciences 113: 7575–7579. 10.1073/pnas.1602205113 PMC4941431 27325781

[bib130] Paredes-Esquivel C , Foronda P, Dunavan CP, Cowie RH. 2023. *Neuroangiostrongyliasis*: Rat lungworm invades Europe. American Journal of Tropical Medicine and Hygiene 108: 857. 36806494 10.4269/ajtmh.22-0782 PMC10077014

[bib131] Patoka J , Bláha M, Kalous L, Vrabec V, Buřič M, Kouba A. 2016. Potential pest transfer mediated by international ornamental plant trade. Scientific Reports 6: 25896. 27221025 10.1038/srep25896 PMC4879528

[bib132] Pereira PC , Parente CE, Carvalho GO, Torres JP, Meire RO, Dorneles PR, Malm O. 2021. A review on pesticides in flower production: A push to reduce human exposure and environmental contamination. Environmental Pollution 289: 117817 34333268 10.1016/j.envpol.2021.117817

[bib133] Petrou KN , Iacovidou E. 2015. Cut-Flower Waste Management. Centre for Environmental Policy, Environmental Quality Research Group. Report no. 75480. www.researchgate.net/publication/360313425.

[bib134] Phelps J , Carrasco LR, Webb EL. 2014. A framework for assessing supply side wildlife conservation. Conservation Biology 28: 244–257. 24471784 10.1111/cobi.12160

[bib135] Pieters B , Hoppenreijs JHT, Beringen R, Sparrius LB, Van Valkenburg JLCH, Velde G, Leuven RSEW. 2018. Risico's van de Sierteeltketen als Introductieroute voor Invasieve Exoten. Nederlandse Voedsel en Warenautoireit. www.nvwa.nl/documenten/plant/planten-in-de-natuur/exoten/risicobeoordelingen/risicobeoordeling-van-de-sierteeltketen-als-introductieroute-voor-invasieve-plantensoorten.

[bib136] Privett S , Bek D, Bailey R, Binns T, Raimondo D, Kirkwood D, Euston-Brown D. 2020. Conservation in the context of wildflower harvesting: The development and implementation of a vulnerability index on the Agulhas Plain of South Africa. Journal of Environmental Planning and Management 63: 1738–1757.

[bib137] Querejeta GA , Ramos LM, Flores AP, Hughes EA, Zalts A, Montserrat JM. 2012. Environmental pesticide distribution in horticultural and floricultural periurban production units. Chemosphere 87: 566–572. 22285036 10.1016/j.chemosphere.2011.12.074

[bib138] Rabobank . 2022 World Floriculture Map 2021. Rabobank. https://research.rabobank.com/far/en/documents/175926_Rabobank_Flower-Map-2021_20211230.pdf.

[bib139] Raitsos DE , Beaugrand G, Georgopoulos D, Zenetos A, Pancucci-Papadopoulou AM, Theocharis A, Papathanassiou E. 2010. Global climate change amplifies the entry of tropical species into the Eastern Mediterranean Sea. Limnology and Oceanography 55: 1478–1484.

[bib140] Rajak P et al. 2023. Agricultural pesticides: Friends or foes to biosphere? Journal of Hazardous Materials Advances 10: 100264.

[bib141] Rato C , Deso G, Renet J, Delaugerre MJ, Marques V, Mochales-Riaño G. 2023. Colonization routes uncovered in a widely introduced Mediterranean gecko, *Tarentola mauritanica*. Scientific Reports 13: 16681. 10.1038/s41598-023-43704-8. 37794160 PMC10551029

[bib142] Raynolds LT. 2012. Fair trade flowers: Global certification, environmental sustainability, and labor standards. Rural Sociology 77: 493–519.

[bib143] Rebelo AD , Bates MF, Burger M, Branch WR, Conradie W. 2019. Range expansion of the common dwarf gecko, *Lygodactylus capensis*: South Africa's most successful reptile invader. Herpetology Notes 12: 643–650.

[bib146] Rowson B , Symondson WOC. 2008. *Selenochlamys ysbryda* sp. Nov. from Wales, UK: A *Testacella*-like slug new to Western Europe (Stylommatophora: Trigonochlamydidae). Journal of Conchology 39: 537–552.

[bib147] Royal Flora Holland . 2023. From July 11 Intensification Inspections *Mandevilla* Destined for United Kingdom. Royal Flora Holland. https://www.royalfloraholland.com/en/news-2023/week-27/from-july-11-intensification-inspections-mandevilla-destined-for-united-kingdom.

[bib148] Sæthre M-G , Staverløkk A, Hågvar EB. 2010 Stowaways in horticultural plants imported from the Netherlands, Germany, and Denmark. Norwegian Journal of Entomology 57: 25–35

[bib149] Scholte EJ , Dijkstra E, Blok H, De Vries A, Takken W, Hofhuis A, Koopmans M, De Boer A, Reusken CBEM. 2008. Accidental importation of the mosquito *Aedes albopictus* into the Netherlands: A survey of mosquito distribution and the presence of dengue virus. Medical and Veterinary Entomology 22: 352–358. 19120963 10.1111/j.1365-2915.2008.00763.x

[bib151] Sharma D. 2008. Current water crisis: Floriculture needs 20 times more water than cotton cultivation. Institute for Agriculture and Trade Policy (18 June 2008). www.iatp.org/news/current-water-crisis-floriculture-needs-20-times-more-water-than-cotton-cultivation.

[bib152] Sherwood D. 2022. *Macrothele calpeiana* (Walckenaer, 1805), an occasional stowaway imported into the United Kingdom with olive trees (Araneae: Macrothelidae). Arachnologische Mitteilungen 44: 59–76.

[bib153] Silva-Rocha I , Salvi D, Sillero N, Mateo JA, Carretero MA. 2015. Snakes on the Balearic Islands: An invasion tale with implications for native biodiversity conservation. PLOS ONE 10: e0121026. 25853711 10.1371/journal.pone.0121026 PMC4390158

[bib154] Sisay MT , Seyoum LA, Seyoum MY. 2017. Correlation study of some physico-chemical parameters and benthic macroinvertebrates metrics on the ecological impacts of floriculture industries along Wedecha River, Debrezeit, Ethiopia. Journal of Coastal Life Medicine 5: 433–440.

[bib155] Staverløkk A , Sæthre M. 2007. Stowaways in Imported Horticultural Plants: Alien and Invasive Species: Assessing Their Bioclimatic Potential in Norway. Bioforsk.

[bib156] Stroud JT , Giery ST, Outerbridge ME. 2017. Establishment of *Anolis sagrei* on Bermuda represents a novel ecological threat to critically endangered Bermuda skinks (*Plestiodon longirostris*). Biological Invasions 19: 1723–1731.

[bib157] Suarez-Lopez JR , Checkoway H, Jacobs DR Jr, Al-Delaimy WK, Gahagan S. 2017. Potential short-term neurobehavioral alterations in children associated with a peak pesticide spray season: The Mother's Day flower harvest in Ecuador. Neurotoxicology 60: 125–133. 28188819 10.1016/j.neuro.2017.02.002 PMC5447476

[bib158] Swinn R. 2017. A comparative LCA of the carbon footprint of cut-flowers: British, Dutch and Kenyan. PhD dissertation, Lancaster University, Lancaster, England, United Kingdom.

[bib159] Tadele M. 2009. Environmental Impacts of Floriculture Industries on Lake Ziway: With Particular Reference to Water Quality. Master's thesis, Addis Ababa University, Addis Adaba, Ethiopia.

[bib160] Tenenbaum D. 2002. Would a rose not smell as sweet? Environmental Health Perspectives 110: A240–A247. 12003767 10.1289/ehp.110-a240 PMC1240850

[bib163] Ticktin T et al. 2023. Wild orchids: A framework for identifying and improving sustainable harvest. Biological Conservation 277: 109816.

[bib164] Timoshyna A , Drinkwater E. 2021. Understanding Corruption Risks in the Global Trade in Wild Plants. Targeting Natural Resources Corruption.

[bib166] Toumi K , Vleminckx C, Van Loco J, Schiffers B. 2016. Pesticide residues on three cut flower species and potential exposure of florists in Belgium. International Journal of Environmental Research and Public Health 13: 943. 27669276 10.3390/ijerph13100943 PMC5086682

[bib165] Toumi K , Joly L, Vleminckx C, Schiffers B. 2017. Risk assessment of florists exposed to pesticide residues through handling of flowers and preparing bouquets. International Journal of Environmental Research and Public Health 14: 526. 10.3390/ijerph14050526. 28505067 PMC5451977

[bib167] Tsimbiri PF , Moturi WN, Sawe J, Henley P, Bend JR. 2015. Health impact of pesticides on residents and horticultural workers in the Lake Naivasha Region, Kenya. Occupational Diseases and Environmental Medicine 3: 24–34. 10.4236/odem.2015.32004.

[bib168] [UNEP] United Nations Environment Programme. 2015. Options for Decoupling Economic Growth from Water Use and Water Pollution: A Report of the International Resource Panel Working Group on Sustainable Water Management. International Resource Panel. www.resourcepanel.org/reports/options-decoupling-economic-growth-water-use-and-water-pollution.

[bib169] Valencia-Montoya WA et al. 2020. Adaptive introgression across semipermeable species boundaries between local *Helicoverpa zea* and invasive *Helicoverpa armigera* moths. Molecular Biology and Evolution 37: 2568–2583. 32348505 10.1093/molbev/msaa108 PMC7475041

[bib170] Van Kleunen M et al. 2018. The changing role of ornamental horticulture in alien plant invasions. Biological Reviews 93: 1421–1437. 29504240 10.1111/brv.12402

[bib171] van Schothorst B , Beriot N, Huerta Lwanga E, Geissen V. 2021. Sources of light density microplastic related to two agricultural practices: The use of compost and plastic mulch. Environments 8: 36.

[bib172] Vilcinskas A. 2015. Pathogens as biological weapons of invasive species. PLOS Pathogens 11: e1004714. 25856550 10.1371/journal.ppat.1004714 PMC4391917

[bib173] Wageningen University and Research . 2023. Developing Product Environmental Footprint Category Rules for Floriculture. Wageningen University and Research. www.wur.nl/en/Research-Results/Research-Institutes/Economic-Research/show-wecr/Developing-Product-Environmental-Footprint-Category-Rules-for-floriculture.htm.

[bib174] Wainwright H , Jordan C, Day H. 2014. Environmental impact of production horticulture. Pages 503–522 in Dixon GR, Aldous DE, eds. Tropical and Subtropical Crops. Springer.

[bib175] Waweru E. 2022. The dark side of the flower sector: The growing exploitation of women in Kenya. Ethical Trading Initiative (10 November 2022). www.ethicaltrade.org/blog/dark-side-flower-sector-growing-exploitation-women-kenya.

[bib177] Williams A. 2007. Comparative Study of Cut Roses for the British Market Produced in Kenya and the Netherlands. Cranfield University. www.fairflowers.de/fileadmin/flp.de/Redaktion/Dokumente/Studien/Comparative_Study_of_Cut_Roses_Feb_2007.pdf.

[bib178] Williams SJ , Jones JPG, Annewandter R, Gibbons JM. 2014. Cultivation can increase harvesting pressure on overexploited plant populations. Ecological Applications 24: 2050–2062. 29188688 10.1890/13-2264.1

[bib179] Wittwer SH. 1993. World-wide use of plastics in horticultural production. HortTechnology 3: 6–19.

[bib180] Wolosin R. 2006. El Milagro de Almeria, España: A Political Ecology of Landscape Change and Greenhouse Agriculture. Master's thesis, University of Montana, Missoula, Montana, United States.

[bib181] World Water Atlas . 2023. More water stress: An ingredient for conflict. World Water Atlas. www.worldwateratlas.org/narratives/drought/water-and-conflict#global-water-stress.

[bib182] Wynberg R. 2023. Biopiracy: Crying wolf or a lever for equity and conservation? Research Policy 52: 104674.

[bib183] Yuan SC , Lekawatana S, Amore TD, Chen FC, Chin SW, Vega DM, Wang YT. 2021. The global orchid market. Pages 1–28 in Chen F-C, Chin S-W, eds. The Orchid Genome. Springer.

[bib184] Zieritz A , Gallardo B, Baker SJ, Britton JR, van Valkenburg JL, Verreycken H, Aldridge DC. 2017. Changes in pathways and vectors of biological invasions in Northwest Europe. Biological Invasions 19: 269–282.

