# Supplementary material for: Understanding the environmental and social risks from the international trade in ornamental plants
Source: Bioscience. 2025 Jan 17;75(3):222–39. doi: 10.1093/biosci/biae124 (PMC11987086; doi:10.1093/biosci/biae124)
Supplement: biae124_Supplemental_File [file biae124_supplemental_file.docx]

***Supplementary information***

**Contents**

Appendix 1: Methods of the trade analysis

Appendix 2: Methods for interception data analysis

Appendix 3: Summary interception data showing number of interceptions by class, and by taxonomic level of reporting

**Appendix 1: Methods of the trade analysis**

We used all data related to live ornamental plants and cut flowers from the TradeMap database, which synthesises customs data from 220 countries and territories, using the Harmonised System (HS) codes (International Trade Centre, 2023). In August 2023, we downloaded all data from 2013-2022 for HS code 06, which encompasses all ‘*Live trees and other plants; bulbs, roots and the like; cut flowers and ornamental foliage*’, to summarise data on key exporters and importers, value of trade, and trends over time. In addition, sub-codes exist for types of ornamental plant products, so we also summarised trade for codes 0601 (bulbs, tubers, rhizomes and other similar plant parts); 0602 (live plants); 0603 (cut flowers); and 0604 (foliage). We used data from each code to calculate the 10 year trends for export value in Figure 1 of the paper, and the top countries for import and export value for Table 1. We then used the ‘country group’ filter to download export value data for Asia, Africa, Latin America and the Caribbean, Europe, North America, and Oceania to produce Figure 2.

Figures were produced from a variety of sources. Figure 3 represents the maximum annual value of export between 2000 and 2021 (Atlas of Economic Complexity 2023). Data was downloaded and processed in Excel to assess the change over time, maximum, minimum and percentage increase in annual export value for each country. Each country was then coded with it’s iso3code and imported into ArcMap 10.8. The table was then connected to a generalised map of the world (ESRI, 2022) using joins and relates on the iso3 field. Maximum export value was then mapped using a continuous scale (though the Netherlands was added separately as the export value was disproportionately higher than all other regions).

For Figure 4, to explore the overall area under floricultural cultivation and the export of various forms of floral commodity (Live plants, bulbs, cut flowers and cut foliage) in 2021. All data was collated from Rabobank 2022a in two separate spreadsheets to provide area under cultivation for floriculture, and transport of various floricultural commodities. Details of the trade of each form of commodity transported by either sea or air was collated into an excel database, listing the country of export, import, the value, and the form of trade. All countries were listed by iso3codes for consistency. Area under cultivation was collated in the same way using iso3codes and Rabobank data. The spreadsheet of area under cultivation was then uploaded into ArcMap 10.8, joined to the generalised world map (ESRI 2022) using joins and relates, then displayed using a continuous colour-ramp. Data on trade directions for each commodity was then uploaded into trademapper (Trademapper 2023) for each commodity in turn, then exported to show traderoutes for each form of commodity. These were then merged with the cultivation area map created in ArcMap to provide both areas under cultivation, and routes of trade.

**References**

Atlas of Economic Complexity (2023) Trees and other plants, live; bulbs, roots and the like; cut flowers and ornamental foliage <https://oec.world/en/profile/hs/live-trees-plants-bulbs-cut-flowers-ornamental-foliage?countryComparisonFlowSelector=Exports>

ESRI (2022) World Countries Generalized <https://hub.arcgis.com/maps/esri::world-countries-generalized>

Trademapper (2023) https://trademapper.co.uk/#

**Appendix 2: Interception data analysi**s

We collaborated with the Netherlands Institute for Vectors, Invasive plants and Plant health (NIVIP), and used interception data for the Netherlands, which was the result of a targeted data collection project (Pieters et al. 2018) on interceptions in 2017 at all ports of entry in the Netherlands. We also obtained UK interception data that reports only notifiable pests (e.g. pest non-compliance), which is available from the Defra portal [Alert List - UK Plant Health Information Portal (defra.gov.uk)](https://planthealthportal.defra.gov.uk/trade/imports/alert-list/).

We cleaned both datasets to remove plants used for food to restrict our analyses to ornamental plants. However, while the split between ornamental and food plants was sometimes clear, with many rows in the Netherlands data labelled as ‘pot plant’ or ‘fruit’, ‘vegetable’ or ‘spices’, others were not straightforward to separate. Many plants are used for both purposes, so we took a conservative approach and removed from the dataset any traded plant or parts of plants that were categorised as food by the data holders, or which were likely to be used primarily for this purpose, such as tomato or cucumber seedlings, or seeds or leaves of basil. For the taxonomic breakdown of pests, see Figure S1.
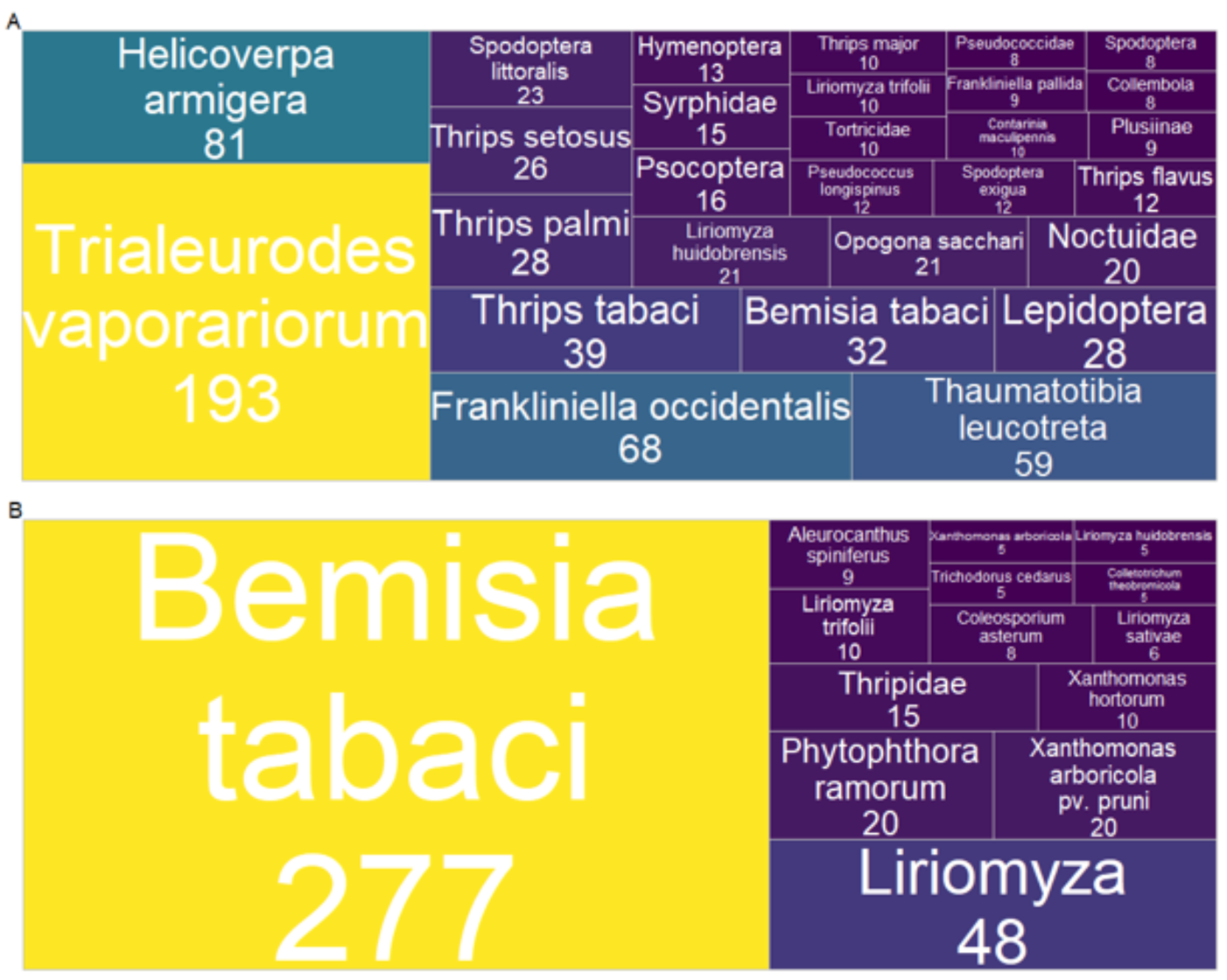


**Figure S1**. Treemap showing the taxonomic breakdown of pest interceptions

We then used Catalogue of Life (Bánki et al. 2023) to check the class and taxonomic level of reporting for each interception. We downloaded all data from the TradeMap (International Trade Centre, 2023) for tons of imports from each exporter to the Netherlands in 2017, and to the UK in the complete years of interception data we had, which was 2021 and 2022. We then calculated the proportions of all interceptions from each country, and all imports from each country, to compare these proportions. We performed a Fishers exact test in R (R Core Team (2024) to compare the imports from and interceptions from each country to the UK 2021-2022, and the Netherlands in 2017. We excluded all countries with less than 1% of both exports or interceptions and any with a value of zero in either of these categories. Results can be seen in tables S1 and S2.

**Table S1.** Summary table showing the number of proportion of plant exports to the UK in 2021-2022 from each exporter, the number and proportion of pest interceptions from each exporting country, and the results of a Fisher’s exact test to compare them.

| Country | Pests detected | All pests detected from other countries | Imports to UK | Imports to UK from all other countries | % of total Pests detected | % total pests detected from other countries | % total imports to UK | % total imports to UK from all other countries | Odds_Ratio | CI_Lower | CI_Upper | p value |
| --- | --- | --- | --- | --- | --- | --- | --- | --- | --- | --- | --- | --- |
| Spain | 2 | 563 | 20208 | 774457 | 0.35 | 99.65 | 2.54 | 97.46 | 0.14 | 1.64E-02 | 4.94E-01 | 0.00 |
| Germany | 3 | 562 | 21949 | 772716 | 0.53 | 99.47 | 2.76 | 97.24 | 0.19 | 3.86E-02 | 5.52E-01 | 0.00 |
| Denmark | 4 | 561 | 20330 | 774335 | 0.71 | 99.29 | 2.56 | 97.44 | 0.27 | 7.38E-02 | 6.99E-01 | 0.00 |
| France | 5 | 560 | 13731 | 780934 | 0.88 | 99.12 | 1.73 | 98.27 | 0.51 | 1.64E-01 | 1.19E+00 | 0.14 |
| Kenya | 14 | 551 | 51267 | 743398 | 2.48 | 97.52 | 6.45 | 93.55 | 0.37 | 2.00E-01 | 6.24E-01 | 0.00 |
| Italy | 29 | 536 | 42374 | 752291 | 5.13 | 94.87 | 5.33 | 94.67 | 0.96 | 6.37E-01 | 1.40E+00 | 0.93 |
| Colombia | 30 | 535 | 29462 | 765203 | 5.31 | 94.69 | 3.71 | 96.29 | 1.46 | 9.73E-01 | 2.10E+00 | 0.06 |
| Netherlands | 244 | 321 | 477546 | 317119 | 43.19 | 56.81 | 60.09 | 39.91 | 0.50 | 4.26E-01 | 5.98E-01 | 0.00 |
| India | 11 | 554 | 890 | 793775 | 1.95 | 98.05 | 0.11 | 99.89 | 17.71 | 8.76E+00 | 3.21E+01 | 0.00 |
| Egypt | 11 | 554 | 1754 | 792911 | 1.95 | 98.05 | 0.22 | 99.78 | 8.98 | 4.45E+00 | 1.62E+01 | 0.00 |
| Malaysia | 15 | 550 | 51 | 794614 | 2.65 | 97.35 | 0.01 | 99.99 | 425.77 | 2.21E+02 | 7.57E+02 | 0.00 |
| Israel | 22 | 543 | 1055 | 793610 | 3.89 | 96.11 | 0.13 | 99.87 | 30.48 | 1.89E+01 | 4.69E+01 | 0.00 |
| Thailand | 69 | 496 | 120 | 794545 | 12.21 | 87.79 | 0.02 | 99.98 | 914.98 | 6.51E+02 | 1.24E+03 | 0.00 |
| Jordan | 1 | 564 | 0 | 794665 | 0.18 | 99.82 | 0.00 | NA | NA | NA | NA | NA |
| Lebanon | 1 | 564 | 0 | 794665 | 0.18 | 99.82 | 0.00 | NA | NA | NA | NA | NA |
| Guinea | 1 | 564 | 0 | 794665 | 0.18 | 99.82 | 0.00 | NA | NA | NA | NA | NA |
| Egypt | 1 | 564 | 1 | 794664 | 0.18 | 99.82 | 0.00 | NA | NA | NA | NA | NA |
| Senegal | 1 | 564 | 2 | 794663 | 0.18 | 99.82 | 0.00 | NA | NA | NA | NA | NA |
| Uganda | 1 | 564 | 502 | 794163 | 0.18 | 99.82 | 0.06 | NA | NA | NA | NA | NA |
| Morocco | 1 | 564 | 3216 | 791449 | 0.18 | 99.82 | 0.40 | NA | NA | NA | NA | NA |
| Portugal | 1 | 564 | 6881 | 787784 | 0.18 | 99.82 | 0.87 | NA | NA | NA | NA | NA |
| Japan | 2 | 563 | 17 | 794648 | 0.35 | 99.65 | 0.00 | NA | NA | NA | NA | NA |
| Tanzania | 2 | 563 | 399 | 794266 | 0.35 | 99.65 | 0.05 | NA | NA | NA | NA | NA |
| Sierra Leone | 3 | 562 | 0 | 794665 | 0.53 | 99.47 | 0.00 | NA | NA | NA | NA | NA |
| Indonesia | 3 | 562 | 16 | 794649 | 0.53 | 99.47 | 0.00 | NA | NA | NA | NA | NA |
| Costa Rica | 3 | 562 | 506 | 794159 | 0.53 | 99.47 | 0.06 | NA | NA | NA | NA | NA |
| Laos | 4 | 561 | 0 | 794665 | 0.71 | 99.29 | 0.00 | NA | NA | NA | NA | NA |
| Vietnam | 4 | 561 | 3 | 794662 | 0.71 | 99.29 | 0.00 | NA | NA | NA | NA | NA |
| Singapore | 5 | 560 | 70 | 794595 | 0.88 | 99.12 | 0.01 | NA | NA | NA | NA | NA |
| Sri Lanka | 5 | 560 | 304 | 794361 | 0.88 | 99.12 | 0.04 | NA | NA | NA | NA | NA |
| Bangladesh | 20 | 545 | 0 | 794665 | 3.54 | 96.46 | 0.00 | NA | NA | NA | NA | NA |
| Nigeria | 38 | 527 | 0 | 794665 | 6.73 | 93.27 | 0.00 | NA | NA | NA | NA | NA |

**Table S2.** Summary table showing the number and proportion of plant exports to the Netherlands in 2017 from each exporter, the number and proportion of pest interceptions from each exporting country, and the results of a Fisher’s exact test to compare them.

| Country | Pests detected | All pests detected from other countries | Imports to NL | Imports to NL from all other countries | % of total pests detected | % total pests detected from other countries | % total imports to NL | % total imports to NL from all other countries | Odds_Ratio | CI_Lower |
| --- | --- | --- | --- | --- | --- | --- | --- | --- | --- | --- |
| Belgium | 1 | 1558 | 356725 | 853272 | 0.06 | 29.48 | 0.00 | 0.00 | 0.01 | 0.00 |
| Germany | 7 | 1552 | 280116 | 929881 | 0.45 | 23.15 | 0.01 | 0.01 | 0.03 | 0.00 |
| Denmark | 1 | 1558 | 49777 | 1160220 | 0.06 | 4.11 | 0.01 | 0.00 | 0.08 | 0.00 |
| France | 1 | 1558 | 24500 | 1185497 | 0.06 | 2.02 | 0.03 | 0.00 | 0.17 | 0.00 |
| Ethiopia | 12 | 1547 | 85480 | 1124517 | 0.77 | 7.06 | 0.10 | 0.05 | 0.18 | 0.00 |
| Spain | 24 | 1535 | 43830 | 1166167 | 1.54 | 3.62 | 0.42 | 0.27 | 0.62 | 0.00 |
| USA | 23 | 1536 | 19293 | 1190704 | 1.48 | 1.59 | 0.92 | 0.58 | 1.39 | 0.84 |
| Ecuador | 28 | 1531 | 11810 | 1198187 | 1.80 | 0.98 | 1.86 | 1.23 | 2.69 | 0.00 |
| Colombia | 21 | 1538 | 6210 | 1203787 | 1.35 | 0.51 | 2.65 | 1.63 | 4.06 | 0.00 |
| China | 54 | 1505 | 23054 | 1186943 | 3.46 | 1.91 | 1.85 | 1.38 | 2.42 | 0.00 |
| Costa Rica | 65 | 1494 | 28800 | 1181197 | 4.17 | 2.38 | 1.78 | 1.37 | 2.29 | 0.00 |
| India | 17 | 1542 | 1627 | 1208370 | 1.09 | 0.13 | 8.19 | 4.75 | 13.20 | 0.00 |
| Tanzania | 29 | 1530 | 4854 | 1205143 | 1.86 | 0.40 | 4.71 | 3.14 | 6.80 | 0.00 |
| Zambia | 30 | 1529 | 5215 | 1204782 | 1.92 | 0.43 | 4.53 | 3.04 | 6.51 | 0.00 |
| Mexico | 26 | 1533 | 2731 | 1207266 | 1.67 | 0.23 | 7.50 | 4.87 | 11.05 | 0.00 |
| Malaysia | 26 | 1533 | 2261 | 1207736 | 1.67 | 0.19 | 9.06 | 5.88 | 13.37 | 0.00 |
| Italy | 21 | 1538 | 80971 | 1129026 | 1.35 | 6.69 | 0.19 | 0.12 | 0.29 | 0.00 |
| Israel | 32 | 1527 | 3511 | 1206486 | 2.05 | 0.29 | 7.20 | 4.90 | 10.23 | 0.00 |
| South Africa | 29 | 1530 | 1702 | 1208295 | 1.86 | 0.14 | 13.46 | 8.95 | 19.48 | 0.00 |
| Thailand | 47 | 1512 | 4601 | 1205396 | 3.01 | 0.38 | 8.14 | 5.95 | 10.90 | 0.00 |
| Uganda | 95 | 1464 | 14144 | 1195853 | 6.09 | 1.17 | 5.49 | 4.41 | 6.76 | 0.00 |
| Kenya | 229 | 1330 | 110326 | 1099671 | 14.69 | 9.12 | 1.72 | 1.48 | 1.98 | 0.00 |
| Zimbabwe | 137 | 1422 | 5406 | 1204591 | 8.79 | 0.45 | 21.46 | 17.85 | 25.63 | 0.00 |
| Rwanda | 14 | 1545 | 624 | 1209373 | NA | NA | NA | NA | NA | NA |
| Honduras | 10 | 1549 | 2495 | 1207502 | NA | NA | NA | NA | NA | NA |
| Sri Lanka | 10 | 1549 | 1777 | 1208220 | NA | NA | NA | NA | NA | NA |
| Japan | 8 | 1551 | 420 | 1209577 | NA | NA | NA | NA | NA | NA |
| Iran | 8 | 1551 | 19 | 1209978 | NA | NA | NA | NA | NA | NA |
| Guatemala | 7 | 1552 | 10189 | 1199808 | NA | NA | NA | NA | NA | NA |
| Portugal | 6 | 1553 | 8584 | 1201413 | NA | NA | NA | NA | NA | NA |
| Slovenia | 6 | 1553 | 529 | 1209468 | NA | NA | NA | NA | NA | NA |
| Brazil | 5 | 1554 | 298 | 1209699 | NA | NA | NA | NA | NA | NA |
| Indonesia | 4 | 1555 | 659 | 1209338 | NA | NA | NA | NA | NA | NA |
| Turkey | 4 | 1555 | 6340 | 1203657 | NA | NA | NA | NA | NA | NA |
| Morocco | 3 | 1556 | 1205 | 1208792 | NA | NA | NA | NA | NA | NA |
| Egypt | 3 | 1556 | 71 | 1209926 | NA | NA | NA | NA | NA | NA |
| Ivory Coast | 3 | 1556 | 3 | 1209994 | NA | NA | NA | NA | NA | NA |
| Ireland | 2 | 1557 | 934 | 1209063 | NA | NA | NA | NA | NA | NA |
| Russia | 2 | 1557 | 42 | 1209955 | NA | NA | NA | NA | NA | NA |
| Vietnam | 2 | 1557 | 376 | 1209621 | NA | NA | NA | NA | NA | NA |
| Chile | 1 | 1558 | 1019 | 1208978 | NA | NA | NA | NA | NA | NA |
| Australia | 1 | 1558 | 274 | 1209723 | NA | NA | NA | NA | NA | NA |
| Austria | 1 | 1558 | 2196 | 1207801 | NA | NA | NA | NA | NA | NA |
| Belarus | 1 | 1558 | 393 | 1209604 | NA | NA | NA | NA | NA | NA |
| El Salvador | 1 | 1558 | 726 | 1209271 | NA | NA | NA | NA | NA | NA |
| New Zealand | 1 | 1558 | 1342 | 1208655 | NA | NA | NA | NA | NA | NA |
| Nigeria | 1 | 1558 | 1 | 1209996 | NA | NA | NA | NA | NA | NA |
| Philippines | 1 | 1558 | 2350 | 1207647 | NA | NA | NA | NA | NA | NA |
| Singapore | 1 | 1558 | 187 | 1209810 | NA | NA | NA | NA | NA | NA |

**References**

Bánki, O., Roskov, Y., Döring, M., Ower, G., Hernández Robles, D. R., Plata Corredor, C. A., Stjernegaard Jeppesen, T., Örn, A., Vandepitte, L., Hobern, D., Schalk, P., DeWalt, R. E., Ma, K., Miller, J., Orrell, T., Aalbu, R., Abbott, J., Adlard, R., Adriaenssens, E. M., et al. (2023). Catalogue of Life Checklist (Version 2023-10-16). Catalogue of Life. <https://doi.org/10.48580/df7lv>

R Core Team (2024). R: A Language and Environment for Statistical Computing_. R Foundation for Statistical Computing, Vienna, Austria. <https://www.R-project.org/>.

**Appendix 3:** Summary interception data showing number of interceptions by class, and by taxonomic level of reporting

| No. | class | country | pest | no | taxonomic_level | Notes |
| --- | --- | --- | --- | --- | --- | --- |
| 37 | Insecta | NL | Trialeurodes vaporariorum | 193 | Species |  |
| 38 | Insecta | NL | Helicoverpa armigera | 81 | Species |  |
| 39 | Insecta | NL | Frankliniella occidentalis | 68 | Species |  |
| 40 | Insecta | NL | Thaumatotibia leucotreta | 59 | Species |  |
| 41 | Insecta | NL | Thrips tabaci | 39 | Species |  |
| 42 | Insecta | NL | Bemisia tabaci | 32 | Species |  |
| 43 | Insecta | NL | Lepidoptera | 28 | Order |  |
| 44 | Insecta | NL | Thrips palmi | 28 | Species |  |
| 45 | Insecta | NL | Thrips setosus | 26 | Species |  |
| 46 | Insecta | NL | Spodoptera littoralis | 23 | Species |  |
| 47 | Insecta | NL | Liriomyza huidobrensis | 21 | Species |  |
| 48 | Insecta | NL | Opogona sacchari | 21 | Species |  |
| 49 | Insecta | NL | Noctuidae | 20 | Family |  |
| 50 | Insecta | NL | Psocoptera | 16 | Order |  |
| 51 | Insecta | NL | Syrphidae | 15 | Family |  |
| 52 | Insecta | NL | Hymenoptera | 13 | Order |  |
| 53 | Insecta | NL | Pseudococcus longispinus | 12 | Species |  |
| 55 | Insecta | NL | Spodoptera exigua | 12 | Species |  |
| 56 | Insecta | NL | Thrips flavus | 12 | Species |  |
| 57 | Insecta | NL | Tortricidae | 10 | Family |  |
| 58 | Insecta | NL | Liriomyza trifolii | 10 | Species |  |
| 59 | Insecta | NL | Thrips major | 10 | Species |  |
| 60 | Insecta | NL | Contarinia maculipennis | 10 | Species |  |
| 61 | Insecta | NL | Plusiinae | 9 | Subfamily | |
| 63 | Insecta | NL | Frankliniella pallida | 9 | Species |  |
| 64 | Insecta | NL | Pseudococcidae | 8 | Family |  |
| 65 | Collembola | NL | Collembola | 8 | Class |  |
| 66 | Insecta | NL | Spodoptera | 8 | Genus |  |
| 67 | Insecta | NL | Planococcus citri | 7 | Species |  |
| 68 | Insecta | NL | Agromyzidae | 7 | Family |  |
| 69 | Insecta | NL | Thrips fuscipennis | 7 | Species |  |
| 70 | Insecta | NL | Diptera | 7 | Order |  |
| 71 | Insecta | NL | Macrosiphum | 7 | Genus |  |
| 72 | Insecta | NL | Singhiella simplex | 7 | Species |  |
| 73 | Arachnida | NL | Aculops fuchsiae | 7 | Species |  |
| 74 | Insecta | NL | Aphididae | 6 | Family |  |
| 75 | Insecta | NL | Frankliniella intonsa | 6 | Species |  |
| 76 | Insecta | NL | Thripinae | 6 | Subfamily | |
| 77 | Insecta | NL | Coleoptera | 6 | Order |  |
| 78 | Arachnida | NL | Neoseiulus californicus | 6 | Species |  |
| 79 | Insecta | NL | Tortrix dinota | 6 | Species |  |
| 80 | Gastropoda | NL | Succineidae | 5 | Family |  |
| 81 | Insecta | NL | Dictyoptera | 5 | Genus |  |
| 82 | Insecta | NL | Pentatomidae | 5 | Family |  |
| 83 | Insecta | NL | Heteroptera | 5 | Suborder |  |
| 84 | Insecta | NL | Cecidomyiidae | 5 | Family |  |
| 85 | Insecta | NL | Haplothrips gowdeyi | 5 | Species |  |
| 86 | Insecta | NL | Geometridae | 4 | Family |  |
| 87 | Insecta | NL | Liriomyza | 4 | Genus |  |
| 88 | Insecta | NL | Aptinothrips rufus | 4 | Species |  |
| 89 | Insecta | NL | Frankliniella schultzei subsp. sulphurea | 4 | Subspecies | |
| 90 | Insecta | NL | Aleurocerus palmae | 4 | Species |  |
| 91 | Insecta | NL | Insecta | 4 | Class |  |
| 92 | Insecta | NL | Pinnaspis aspidistrae | 4 | Species |  |
| 93 | Insecta | NL | Empoasca | 4 | Genus |  |
| 94 | Insecta | NL | Limothrips cerealium | 4 | Species |  |
| 95 | Arachnida | NL | Oribatida | 4 | Superorder | |
| 96 | Arachnida | NL | Tetranychus urticae | 4 | Species |  |
| 97 | Arachnida | NL | Neoseiulus cucumeris | 4 | Species |  |
| 98 | Insecta | NL | Cameraria gaultheriella | 4 | Species |  |
| 99 | Insecta | NL | Cinara | 4 | Genus |  |
| 100 | Insecta | NL | Adelgidae | 4 | Family |  |
| 101 | Insecta | NL | Gynaikothrips uzeli | 4 | Species |  |
| 102 | Insecta | NL | Ctenarytaina peregrina | 4 | Species |  |
| 103 | Insecta | NL | Siphoninus phillyreae | 4 | Species |  |
| 104 | Insecta | NL | Aleuroclava jasmini | 4 | Species |  |
| 105 | Insecta | NL | Spodoptera frugiperda | 3 | Species |  |
| 106 | Insecta | NL | Aeolothrips intermedius | 3 | Species |  |
| 107 | Unclear | NL | Coccoidea | 3 | Unclear | could be genus of fungi or superfamily in insecta |
| 108 | Insecta | NL | Hemiberlesia cyanophylli | 3 | Species |  |
| 109 | Insecta | NL | Stratiomyidae | 3 | Family |  |
| 110 | Insecta | NL | Pentatomoidea | 3 | Superfamily | |
| 111 | Insecta | NL | Coreidae | 3 | Family |  |
| 112 | Insecta | NL | Pinnaspis strachani | 3 | Species |  |
| 113 | Insecta | NL | Rhynchophorus ferrugineus | 3 | Species |  |
| 114 | Insecta | NL | Staphylinidae | 3 | Family |  |
| 115 | Insecta | NL | Ripersiella multiporifera | 3 | Species |  |
| 116 | Insecta | NL | Ichneumonidae | 3 | Family |  |
| 117 | Insecta | NL | Aeolothrips scabiosatibia | 3 | Species |  |
| 118 | Insecta | NL | Lygaeidae | 3 | Family |  |
| 119 | Insecta | NL | Sciaridae | 3 | Family |  |
| 120 | Insecta | NL | Lasius niger | 3 | Species |  |
| 121 | Insecta | NL | Cacoecimorpha pronubana | 3 | Species |  |
| 122 | Symphypleona | NL | Sminthurus viridis | 3 | Species |  |
| 123 | Insecta | NL | Cortinicara gibbosa | 3 | Species |  |
| 124 | Insecta | NL | Orthoperus | 3 | Genus |  |
| 125 | Arachnida | NL | Rhizoglyphus robini | 3 | Species |  |
| 126 | Insecta | NL | Coccotrypes cyperi | 3 | Species |  |
| 127 | Insecta | NL | Trilocha varians | 3 | Species |  |
| 128 | Insecta | NL | Ctenarytaina eucalypti | 3 | Species |  |
| 129 | Insecta | NL | Phyllonorycter platani | 3 | Species |  |
| 130 | NA | NL | NA | 3 | NA | No name included |
| 131 | Insecta | NL | Dialeurodes citri | 3 | Species |  |
| 132 | Insecta | NL | Lamiini | 3 | Tribe |  |
| 133 | Insecta | NL | Crepidodera | 3 | Genus |  |
| 134 | Insecta | NL | Aphis fabae | 2 | Species |  |
| 135 | Insecta | NL | Chalcidoidea | 2 | Superfamily | |
| 136 | Arachnida | NL | Panonychus citri | 2 | Species |  |
| 137 | Arachnida | NL | Tydeidae | 2 | Family |  |
| 138 | Insecta | NL | Coccus hesperidum | 2 | Species |  |
| 139 | Insecta | NL | Elacatis | 2 | Genus |  |
| 140 | Insecta | NL | Exophthalmus jekelianus | 2 | Species |  |
| 141 | Insecta | NL | Aphis | 2 | Genus |  |
| 142 | Insecta | NL | Aphis gossypii | 2 | Species |  |
| 143 | Insecta | NL | Chaetanaphothrips orchidii | 2 | Species |  |
| 144 | Arachnida | NL | Salticidae | 2 | Family |  |
| 145 | Gastropoda | NL | Gastropoda | 2 | Class |  |
| 146 | Insecta | NL | Aspidiotus destructor | 2 | Species |  |
| 147 | Chromadorea | NL | Helicotylenchus | 2 | Genus |  |
| 148 | Insecta | NL | Protapion fulvipes | 2 | Species |  |
| 149 | Insecta | NL | Scymnus | 2 | Genus |  |
| 150 | Insecta | NL | Tipulidae | 2 | Family |  |
| 151 | Insecta | NL | Taeniothrips eucharii | 2 | Species |  |
| 152 | Insecta | NL | Ozodes multituberculatus | 2 | Species |  |
| 153 | Insecta | NL | Karnyothrips melaleucus | 2 | Species |  |
| 154 | Insecta | NL | Aleyrodidae | 2 | Family |  |
| 155 | Arachnida | NL | Tetranychidae | 2 | Family |  |
| 156 | Insecta | NL | Thrips nigropilosus | 2 | Species |  |
| 157 | Arachnida | NL | Typhlodromips swirskii | 2 | Species |  |
| 158 | Insecta | NL | Aulacorthum solani | 2 | Species |  |
| 159 | Insecta | NL | Necrobia violacea | 2 | Species |  |
| 160 | Insecta | NL | Trypeta immaculata | 2 | Species |  |
| 161 | Insecta | NL | Cossus cossus | 2 | Species |  |
| 162 | Insecta | NL | Linepithema humile | 2 | Species |  |
| 163 | Arachnida | NL | Meyerius | 2 | Genus |  |
| 164 | Insecta | NL | Oecophoridae | 2 | Family |  |
| 165 | Diplopoda | NL | Polyxenus | 2 | Genus |  |
| 166 | Insecta | NL | Frankliniella schultzei subsp. schultzei | 2 | Subspecies | |
| 167 | Insecta | NL | Harpalini | 2 | Tribe |  |
| 168 | Insecta | NL | Kalotermitidae | 2 | Family |  |
| 169 | Arachnida | NL | Pseudoscorpiones | 2 | Order |  |
| 170 | Insecta | NL | Orius minutus | 2 | Species |  |
| 171 | Insecta | NL | Bryocoris pteridis | 2 | Species |  |
| 172 | Arachnida | NL | Cunaxidae | 2 | Family |  |
| 173 | Insecta | NL | Nysius ericae | 2 | Species |  |
| 174 | Arachnida | NL | Ornithonyssus sylviarum | 2 | Species |  |
| 175 | Insecta | NL | Oxythrips ajugae | 2 | Species |  |
| 176 | Insecta | NL | Cupressatia siskiyou | 2 | Species |  |
| 177 | Insecta | NL | Eulophidae | 2 | Family |  |
| 178 | Symphypleona | NL | Sminthuridae | 2 | Family |  |
| 179 | Insecta | NL | Carpophilus nepos | 2 | Species |  |
| 180 | Insecta | NL | Nanophyes marmoratus | 2 | Species |  |
| 181 | Insecta | NL | Trialeurodes | 2 | Genus |  |
| 182 | Insecta | NL | Ferrisia virgata | 2 | Species |  |
| 183 | Insecta | NL | Odontothrips karnyi | 2 | Species |  |
| 184 | Insecta | NL | Scolytus intricatus | 2 | Species |  |
| 185 | Insecta | NL | Epuraea | 2 | Genus |  |
| 186 | Insecta | NL | Uleiota planata | 2 | Species |  |
| 187 | Insecta | NL | Aphidoidea | 2 | Superfamily | |
| 188 | Insecta | NL | Lestodiplosini | 2 | Tribe |  |
| 189 | Insecta | NL | Neomyzus | 2 | Genus |  |
| 190 | Arachnida | NL | Mesostigmata | 2 | Order |  |
| 191 | Arachnida | NL | Rhodacaridae | 2 | Family |  |
| 192 | Arachnida | NL | Tyrophagus | 2 | Genus | Not in CoL |
| 193 | Insecta | NL | Caloptilia roscipennella | 2 | Species |  |
| 194 | Insecta | NL | Rhagoletis completa | 2 | Species |  |
| 195 | Insecta | NL | Agrotis segetum | 2 | Species |  |
| 196 | Insecta | NL | Frankliniella schultzei | 2 | Species |  |
| 197 | Insecta | NL | Aeolothrips tenuicornis | 2 | Species |  |
| 198 | Insecta | NL | Gynaikothrips ficorum | 2 | Species |  |
| 199 | Insecta | NL | Josephiella | 2 | Genus |  |
| 200 | Insecta | NL | Lascoria orneodalis | 2 | Species |  |
| 201 | Insecta | NL | Stictoleptura rubra | 2 | Species |  |
| 202 | Insecta | NL | Leptocybe invasa | 2 | Species |  |
| 203 | Insecta | NL | Ophelimus maskelli | 2 | Species |  |
| 204 | Insecta | NL | Gonipterus | 2 | Genus |  |
| 205 | Insecta | NL | Olethreutinae | 2 | Subfamily | |
| 206 | Insecta | NL | Thrips | 2 | Genus |  |
| 207 | Insecta | NL | Clytus arietis | 2 | Species |  |
| 208 | Insecta | NL | Euphyllura olivina | 2 | Species |  |
| 209 | Insecta | NL | Limoniidae | 2 | Family |  |
| 210 | Insecta | NL | Monochamus | 2 | Genus |  |
| 211 | Insecta | NL | Arhopalus rusticus | 2 | Species |  |
| 212 | Insecta | NL | Cosmia trapezina | 2 | Species |  |
| 213 | Arachnida | NL | Epitrimerus serbicus | 2 | Species |  |
| 214 | Arachnida | NL | Tarsonemus | 2 | Genus |  |
| 215 | Arachnida | NL | Tarsonemus lacustris | 2 | Species |  |
| 216 | Insecta | NL | Helicoverpa | 2 | Genus |  |
| 217 | Insecta | NL | Eriosoma lanigerum | 2 | Species |  |
| 218 | Insecta | NL | Metopolophium | 2 | Genus |  |
| 219 | Insecta | NL | Spodoptera litura | 2 | Species |  |
| 220 | Arachnida | NL | Tetranychus | 2 | Genus |  |
| 221 | Insecta | NL | Arge pagana | 2 | Species |  |
| 222 | Insecta | NL | Harmonia quadripunctata | 2 | Species |  |
| 223 | Collembola | NL | Entomobrya | 2 | Genus |  |
| 224 | Insecta | NL | Lygus rugulipennis | 2 | Species |  |
| 225 | Insecta | NL | Myzaphis rosarum | 2 | Species |  |
| 226 | Insecta | NL | Aleurocanthus camelliae | 2 | Species |  |
| 227 | Insecta | NL | Vanessa atalanta | 2 | Species |  |
| 228 | Insecta | NL | Thrips urticae | 2 | Species |  |
| 229 | Insecta | NL | Tabanidae | 1 | Family |  |
| 230 | Insecta | NL | Chrysodeixis chalcites | 1 | Species |  |
| 231 | Insecta | NL | Athalia rosae | 1 | Species |  |
| 232 | Insecta | NL | Semiaphis | 1 | Genus |  |
| 233 | Magnoliopsida | NL | Hydrocotyle | 1 | Genus |  |
| 234 | Insecta | NL | Amyna punctum | 1 | Species |  |
| 235 | Arachnida | NL | Amblyseius obtusus | 1 | Species |  |
| 236 | Arachnida | NL | Neoseiulus alpinus | 1 | Species |  |
| 237 | Insecta | NL | Depressaria radiella | 1 | Species |  |
| 238 | Insecta | NL | Neomyzus circumflexum | 1 | Species |  |
| 239 | Insecta | NL | Coccidae | 1 | Family |  |
| 240 | Insecta | NL | Phylus | 1 | Genus |  |
| 241 | Insecta | NL | Diaspis boisduvalii | 1 | Species |  |
| 242 | Insecta | NL | Frankliniella williamsi | 1 | Species |  |
| 243 | Insecta | NL | Protopulvinaria pyriformis | 1 | Species |  |
| 244 | Insecta | NL | Saissetia | 1 | Genus |  |
| 245 | Insecta | NL | Saissetia coffeae | 1 | Species |  |
| 246 | Insecta | NL | Issus coleoptratus | 1 | Species |  |
| 247 | Insecta | NL | Grammoptera ruficornis | 1 | Species |  |
| 248 | Lecanoromycetes | NL | Xyleborus | 1 | Genus |  |
| 249 | Insecta | NL | Ceroplastes stellifer | 1 | Species |  |
| 250 | Arachnida | NL | Pseudoscorpionida | 1 | Order | Synonym |
| 251 | Insecta | NL | Hypercompe icasia | 1 | Species |  |
| 252 | Insecta | NL | Anastrepha | 1 | Genus |  |
| 253 | Insecta | NL | Spodoptera latifascia | 1 | Species |  |
| 254 | Insecta | NL | Baridinae | 1 | Subfamily | |
| 255 | Insecta | NL | Castolus trinotatus | 1 | Species |  |
| 256 | Insecta | NL | Cleistolophus viridimargo | 1 | Species |  |
| 257 | Insecta | NL | Notodontidae | 1 | Family |  |
| 258 | Insecta | NL | Eleodes | 1 | Genus |  |
| 259 | Arachnida | NL | Rhipicephalus | 1 | Genus |  |
| 260 | Insecta | NL | Icerya purchasi | 1 | Species |  |
| 261 | Insecta | NL | Acutaspis albopicta | 1 | Species |  |
| 262 | Insecta | NL | Ceroplastes | 1 | Genus |  |
| 263 | Insecta | NL | Orthoptera | 1 | Order |  |
| 264 | Chilopoda | NL | Chilopoda | 1 | Class |  |
| 265 | Insecta | NL | Diplopseustis perieresalis | 1 | Species |  |
| 266 | Chromadorea | NL | Meloidogyne | 1 | Genus |  |
| 267 | Chromadorea | NL | Paratylenchus | 1 | Genus |  |
| 268 | Chromadorea | NL | Pratylenchus penetrans | 1 | Species |  |
| 269 | Insecta | NL | Aspidiotus nerii | 1 | Species |  |
| 270 | Arachnida | NL | Proprioseiopsis mexicanus | 1 | Species |  |
| 271 | Insecta | NL | Apocrita | 1 | Suborder | Not in CoL |
| 272 | Insecta | NL | Chrysomphalus aonidum | 1 | Species |  |
| 273 | Insecta | NL | Crematogaster | 1 | Genus |  |
| 274 | Insecta | NL | Erebidae | 1 | Family |  |
| 275 | Insecta | NL | Saperdini | 1 | Tribe |  |
| 276 | Insecta | NL | Tettigoniidae | 1 | Family |  |
| 277 | Insecta | NL | Elateridae | 1 | Family |  |
| 278 | Insecta | NL | Otiorhynchus sulcatus | 1 | Species |  |
| 279 | Insecta | NL | Europs | 1 | Genus |  |
| 280 | Insecta | NL | Silvanus muticus | 1 | Species |  |
| 281 | Insecta | NL | Ileomus mucoreus | 1 | Species |  |
| 282 | Insecta | NL | Technomyrmex albipes | 1 | Species |  |
| 283 | Arachnida | NL | Parasitus americanus | 1 | Species |  |
| 284 | Insecta | NL | Hercinothrips | 1 | Genus |  |
| 285 | Diplopoda | NL | Oxidus | 1 | Genus |  |
| 286 | Insecta | NL | Dasyproctus bipunctatus | 1 | Species |  |
| 287 | Gastropoda | NL | Deroceras | 1 | Genus |  |
| 288 | Insecta | NL | Haplothrips nigricornis | 1 | Species |  |
| 289 | Insecta | NL | Ephemeroptera | 1 | Order |  |
| 290 | Insecta | NL | Spodoptera cosmioides | 1 | Species |  |
| 291 | Insecta | NL | Pyralidae | 1 | Family |  |
| 292 | Insecta | NL | Psychodidae | 1 | Family |  |
| 293 | Insecta | NL | Cercopis | 1 | Genus |  |
| 294 | Insecta | NL | Deltocephalinae | 1 | Subfamily | |
| 295 | Insecta | NL | Ctenoplusia limbirena | 1 | Species |  |
| 296 | Insecta | NL | Microlepidoptera | 1 | NA - paraphyletic | |
| 297 | Insecta | NL | Phenacoccus madeirensis | 1 | Species |  |
| 298 | Insecta | NL | Thysanoplusia orichalcea | 1 | Species |  |
| 299 | Arachnida | NL | Acalitus brevitarsus | 1 | Species |  |
| 300 | Insecta | NL | Aeolothrips versicolor | 1 | Species |  |
| 301 | Insecta | NL | Anaphothrips obscurus | 1 | Species |  |
| 302 | Insecta | NL | Mycterothrips salicis | 1 | Species |  |
| 303 | Insecta | NL | Thrips alni | 1 | Species |  |
| 304 | Insecta | NL | Ulopa reticulata | 1 | Species |  |
| 305 | Insecta | NL | Rhaphigaster nebulosa | 1 | Species |  |
| 306 | Insecta | NL | Mamestra brassicae | 1 | Species |  |
| 307 | Insecta | NL | Eysarcoris | 1 | Genus |  |
| 308 | Insecta | NL | Plutella xylostella | 1 | Species |  |
| 309 | Insecta | NL | Ceutorhynchinae | 1 | Subfamily | not in CoL |
| 310 | Insecta | NL | Ceutorhynchini | 1 | Tribe | not in CoL |
| 311 | Arachnida | NL | Chelifer cancroides | 1 | Species |  |
| 312 | Insecta | NL | Lymantriidae | 1 | Subfamily | not in CoL |
| 313 | Insecta | NL | Nitidulidae | 1 | Family |  |
| 314 | Insecta | NL | Tenothrips | 1 | Genus |  |
| 315 | Arachnida | NL | Balaustium | 1 | Genus |  |
| 316 | Insecta | NL | Rhizoecus keysensis | 1 | Species |  |
| 317 | Insecta | NL | Ripersiella planetica | 1 | Species |  |
| 318 | Insecta | NL | Crambidae | 1 | Family |  |
| 319 | Insecta | NL | Agelastica alni | 1 | Species |  |
| 320 | Insecta | NL | Haplothrips setiger | 1 | Species |  |
| 321 | Insecta | NL | Frankliniella panamensis | 1 | Species |  |
| 322 | Insecta | NL | Copitarsia | 1 | Genus |  |
| 323 | Insecta | NL | Ahasverus advena | 1 | Species |  |
| 324 | Insecta | NL | Copitarsia corruda | 1 | Species |  |
| 325 | Insecta | NL | Myrmicinae | 1 | Subfamily | |
| 326 | Insecta | NL | Peridroma saucia | 1 | Species |  |
| 327 | Insecta | NL | Pieridae | 1 | Family |  |
| 328 | Insecta | NL | Mythimna | 1 | Genus |  |
| 329 | Insecta | NL | Planococcus minor | 1 | Species |  |
| 330 | Insecta | NL | Eriosoma | 1 | Genus |  |
| 331 | Insecta | NL | Pemphigus | 1 | Genus |  |
| 332 | Insecta | NL | Pseudococcus viburni | 1 | Species |  |
| 333 | Insecta | NL | Phloeosinus bicolor | 1 | Species |  |
| 334 | Insecta | NL | Aulacaspis yasumatsui | 1 | Species |  |
| 335 | Insecta | NL | Carpophilus dimidiatus | 1 | Species |  |
| 336 | Insecta | NL | Anoecia caricis | 1 | Species |  |
| 337 | Insecta | NL | Adalia bipunctata | 1 | Species |  |
| 338 | Insecta | NL | Eupteryx | 1 | Genus |  |
| 339 | Insecta | NL | Vesiculaphis | 1 | Genus |  |
| 340 | Liliopsida | NL | Cyperus esculentus | 1 | Species |  |
| 341 | Insecta | NL | Trialeurodes fernaldi | 1 | Species |  |
| 342 | Insecta | NL | Glaucothrips glaucus | 1 | Species |  |
| 343 | Gastropoda | NL | Ariolimax columbianus | 1 | Species |  |
| 344 | Arachnida | NL | Oligonychus ilicis | 1 | Species |  |
| 345 | Insecta | NL | Parthenolecanium persicae | 1 | Species |  |
| 346 | Insecta | NL | Philephedra tuberculosa | 1 | Species |  |
| 347 | Insecta | NL | Acizzia uncatoides | 1 | Species |  |
| 348 | Insecta | NL | Thrips meridionalis | 1 | Species |  |
| 349 | Insecta | NL | Lathyromyza schlechtendali | 1 | Species |  |
| 350 | Insecta | NL | Lestodiplosis | 1 | Genus |  |
| 351 | Insecta | NL | Contarinia medicaginis | 1 | Species |  |
| 352 | Insecta | NL | Cardiocondyla obscurior | 1 | Species |  |
| 353 | Insecta | NL | Technomyrmex vitiensis | 1 | Species |  |
| 354 | Insecta | NL | Curculionidae | 1 | Family |  |
| 355 | Insecta | NL | Agrilus angustulus | 1 | Species |  |
| 356 | Insecta | NL | Polydrusus formosus | 1 | Species |  |
| 357 | Insecta | NL | Phymatodes | 1 | Genus |  |
| 358 | Insecta | NL | Pyrrhidium sanguineum | 1 | Species |  |
| 359 | Insecta | NL | Mycetophagidae | 1 | Family |  |
| 360 | Insecta | NL | Liriomyza bryoniae | 1 | Species |  |
| 361 | Insecta | NL | Nasonovia ribisnigri | 1 | Species |  |
| 362 | Insecta | NL | Technomyrmex | 1 | Genus |  |
| 363 | Gastropoda | NL | Amerianna | 1 | Genus |  |
| 364 | Arachnida | NL | Anystis baccarum | 1 | Species |  |
| 365 | Arachnida | NL | Linyphiidae | 1 | Family |  |
| 366 | Arachnida | NL | Neoseiulus barkeri | 1 | Species |  |
| 367 | Arachnida | NL | Tetranychus kanzawai | 1 | Species |  |
| 368 | Insecta | NL | Dysaphis tulipae | 1 | Species |  |
| 369 | Arachnida | NL | Histiostoma | 1 | Genus |  |
| 370 | Arachnida | NL | Aceria erinea | 1 | Species |  |
| 371 | Insecta | NL | Anthribus fasciatus | 1 | Species |  |
| 372 | Insecta | NL | Phycitini | 1 | Tribe |  |
| 373 | Insecta | NL | Drosophilidae | 1 | Family |  |
| 374 | Arachnida | NL | Aceria tristriata | 1 | Species |  |
| 375 | Insecta | NL | Eupteryx decemnotata | 1 | Species |  |
| 376 | Insecta | NL | Frankliniella borinquen | 1 | Species |  |
| 377 | Insecta | NL | Sitophilus zeamais | 1 | Species |  |
| 378 | Insecta | NL | Thrips origani | 1 | Species |  |
| 379 | Insecta | NL | Pyrausta | 1 | Genus |  |
| 380 | Insecta | NL | Corticariinae | 1 | Subfamily | |
| 381 | Insecta | NL | Trioza alacris | 1 | Species |  |
| 382 | Insecta | NL | Anomala dubia | 1 | Species |  |
| 383 | Arachnida | NL | Tyrophagus putrescentiae | 1 | Species |  |
| 384 | Insecta | NL | Parapoynx | 1 | Genus |  |
| 385 | Insecta | NL | Hoplandrothrips flavipes | 1 | Species |  |
| 386 | Insecta | NL | Horidiplosis ficifolii | 1 | Species |  |
| 387 | Insecta | NL | Josephiella microcarpae | 1 | Species |  |
| 388 | Insecta | NL | Noctuoidea | 1 | Superfamily | |
| 389 | Insecta | NL | Phratora | 1 | Genus |  |
| 390 | Insecta | NL | Sesiidae | 1 | Family |  |
| 391 | Insecta | NL | Androthrips ramachandrai | 1 | Species |  |
| 392 | Insecta | NL | Olenecamptus | 1 | Genus |  |
| 393 | Insecta | NL | Pseudaulacaspis cockerelli | 1 | Species |  |
| 394 | Arachnida | NL | Arachnida | 1 | Class |  |
| 395 | Insecta | NL | Ceratocorema | 1 | Genus |  |
| 396 | Insecta | NL | Ceratocorema cymbalistis | 1 | Species |  |
| 397 | Insecta | NL | Choreutidae | 1 | Family |  |
| 398 | Insecta | NL | Gracillariidae | 1 | Family |  |
| 399 | Insecta | NL | Leptocentrus taurus | 1 | Species |  |
| 400 | Insecta | NL | Closterocerus chamaeleon | 1 | Species |  |
| 401 | Insecta | NL | Cerambycidae | 1 | Family |  |
| 402 | Insecta | NL | Diarsia rubi | 1 | Species |  |
| 403 | Insecta | NL | Hypeninae | 1 | Subfamily | |
| 404 | Insecta | NL | Miridae | 1 | Family |  |
| 405 | Insecta | NL | Lantanophaga pusillidactyla | 1 | Species |  |
| 406 | Insecta | NL | Phenacoccus solenopsis | 1 | Species |  |
| 407 | Insecta | NL | Cacopsylla fulguralis | 1 | Species |  |
| 408 | Insecta | NL | Monochamus sartor | 1 | Species |  |
| 409 | Insecta | NL | Otiorhynchus apenninus | 1 | Species |  |
| 410 | Insecta | NL | Pulvinaria floccifera | 1 | Species |  |
| 411 | Insecta | NL | Pulvinaria hydrangeae | 1 | Species |  |
| 412 | Insecta | NL | Scirtothrips dorsalis | 1 | Species |  |
| 413 | Insecta | NL | Auchenorrhyncha | 1 | Suborder |  |
| 414 | Insecta | NL | Diaspididae | 1 | Family |  |
| 415 | Insecta | NL | Dendrothrips ornatus | 1 | Species |  |
| 416 | Arachnida | NL | Amblyseius andersoni | 1 | Species |  |
| 417 | Arachnida | NL | Brevipalpus essigi | 1 | Species |  |
| 418 | Arachnida | NL | Euseius finlandicus | 1 | Species |  |
| 419 | Arachnida | NL | Paraseiulus triporus | 1 | Species |  |
| 420 | Insecta | NL | Dichromothrips corbetti | 1 | Species |  |
| 421 | Insecta | NL | Haplothrips | 1 | Genus |  |
| 422 | Insecta | NL | Acrida | 1 | Genus |  |
| 423 | Arachnida | NL | Araneae | 1 | Order |  |
| 424 | Insecta | NL | Contarinia | 1 | Genus |  |
| 425 | Collembola | NL | Lepidocyrtus | 1 | Genus |  |
| 426 | Insecta | NL | Nematocera | 1 | Genus |  |
| 427 | Insecta | NL | Oryzaephilus mercator | 1 | Species |  |
| 428 | Insecta | NL | Tapinoma melanocephalum | 1 | Species |  |
| 429 | Arachnida | NL | Theridiidae | 1 | Family |  |
| 430 | Insecta | NL | Cerataphis | 1 | Genus |  |
| 431 | Insecta | NL | Cerataphis orchidearum | 1 | Species |  |
| 432 | Insecta | NL | Culicidae | 1 | Family |  |
| 433 | Insecta | NL | Cinara pilicornis | 1 | Species |  |
| 434 | Insecta | NL | Neodiprion sertifer | 1 | Species |  |
| 435 | Insecta | NL | Adelges | 1 | Genus |  |
| 436 | Insecta | NL | Diprion pini | 1 | Species |  |
| 437 | Insecta | NL | Chlorophorus annularis | 1 | Species |  |
| 438 | Insecta | NL | Otiorhynchus | 1 | Genus |  |
| 439 | Arachnida | NL | Stigmaeopsis nanjingensis | 1 | Species |  |
| 440 | Arachnida | NL | Schizotetranychus bambusae | 1 | Species |  |
| 441 | Insecta | NL | Phycitinae | 1 | Subfamily | |
| 442 | Arachnida | NL | Neoseiulus | 1 | Genus |  |
| 443 | Insecta | NL | Stereomita andropogonis | 1 | Species |  |
| 444 | Arachnida | NL | Typhlodromus capparidis | 1 | Species |  |
| 445 | Insecta | NL | Tropidothorax leucopterus | 1 | Species |  |
| 446 | Insecta | NL | Fiorinia theae | 1 | Species |  |
| 447 | Insecta | NL | Orius | 1 | Genus |  |
| 448 | Arachnida | NL | Eupodidae | 1 | Family |  |
| 449 | Arachnida | NL | Xenotarsonemus | 1 | Genus |  |
| 450 | Insecta | NL | Gastrophysa viridula | 1 | Species |  |
| 451 | Arachnida | NL | Trachytes aegrota | 1 | Species |  |
| 452 | Insecta | NL | Philophylla caesio | 1 | Species |  |
| 453 | Insecta | NL | Typhlocybinae | 1 | Subfamily | |
| 454 | Insecta | NL | Cartodere bifasciata | 1 | Species |  |
| 455 | Insecta | NL | Chirothrips | 1 | Genus |  |
| 456 | Insecta | NL | Anoplophora chinensis | 1 | Species |  |
| 457 | Insecta | NL | Paraswammerdamia nebulella | 1 | Species |  |
| 458 | Insecta | NL | Zygina flammigera | 1 | Species |  |
| 459 | Insecta | NL | Archipini | 1 | Tribe |  |
| 460 | NA | NL | Arthropoda | 1 | Phylum |  |
| 461 | Insecta | NL | Atrichelaphinis | 1 | Genus |  |
| 462 | Insecta | NL | Aulacorthum | 1 | Genus |  |
| 463 | Insecta | NL | Choristoneura occidentalis | 1 | Species |  |
| 464 | Insecta | NL | Craspedothrips | 1 | Genus |  |
| 465 | Insecta | NL | Evania | 1 | Genus |  |
| 466 | Insecta | NL | Macrosiphum euphorbiae | 1 | Species |  |
| 467 | Insecta | NL | Medythia quaterna | 1 | Species |  |
| 468 | Insecta | NL | Planococcus | 1 | Genus |  |
| 469 | Insecta | NL | Reduviidae | 1 | Family |  |
| 470 | Insecta | NL | Trialeurodes abutiloneus | 1 | Species |  |
| 471 | Insecta | NL | Anaphothrips sudanensis | 1 | Species |  |
| 472 | Insecta | NL | Haplothrips articulosus | 1 | Species |  |
| 473 | Insecta | NL | Haplothrips ganglbaueri | 1 | Species |  |
| 474 | Arachnida | NL | Kampimodromus aberrans | 1 | Species |  |
| 475 | Arachnida | NL | Phytoseiulus persimilis | 1 | Species |  |
| 476 | Insecta | NL | Thrips cacuminis | 1 | Species |  |
| 477 | Insecta | NL | Echinothrips americanus | 1 | Species |  |
| 478 | Diplopoda | NL | Paradoxosomatidae | 1 | Family |  |
| 479 | Insecta | NL | Aleurothrixus floccosus | 1 | Species |  |
| 480 | Insecta | NL | Aphis nerii | 1 | Species |  |
| 481 | Insecta | NL | Phyllocnistis citrella | 1 | Species |  |
| 482 | Arachnida | NL | Tyrophagus neiswanderi | 1 | Species |  |
| 483 | Insecta | NL | Chrysomela populi | 1 | Species |  |
| 484 | Insecta | NL | Pseudaulacaspis pentagona | 1 | Species |  |
| 485 | Insecta | NL | Frankliniella | 1 | Genus |  |
| 486 | Arachnida | NL | Aceria kuko | 1 | Species |  |
| 487 | Insecta | NL | Chrysopidae | 1 | Family |  |
| 488 | Insecta | NL | Mycetophilidae | 1 | Family |  |
| 489 | Insecta | NL | Pseudaonidia paeoniae | 1 | Species |  |
| 490 | Insecta | NL | Scarabaeidae | 1 | Family |  |
| 491 | Insecta | NL | Aleurotuba jelinekii | 1 | Species |  |
| 492 | Insecta | NL | Massilieurodes setiger | 1 | Species |  |
| 493 | Gastropoda | NL | Deroceras laeve | 1 | Species |  |
| 494 | Insecta | NL | Heliothrips haemorrhoidalis | 1 | Species |  |
| 495 | Arachnida | NL | Colomerus vitis | 1 | Species |  |
| 496 | Insecta | NL | Anobium punctatum | 1 | Species |  |
| 497 | Insecta | NL | Agromyza | 1 | Genus |  |
| 1 | Insecta | UK | Bemisia tabaci | 277 | Species |  |
| 5 | Insecta | UK | Liriomyza | 48 | Genus |  |
| 2 | Peronosporea | UK | Phytophthora ramorum | 20 | Species |  |
| 11 | Xanthomodales | UK | Xanthomonas arboricola pv. pruni | 20 | Strain |  |
| 9 | Insecta | UK | Thripidae | 15 | Family |  |
| 19 | Xanthomodales | UK | Xanthomonas hortorum | 10 | Species |  |
| 24 | Insecta | UK | Liriomyza trifolii | 10 | Species |  |
| 12 | Insecta | UK | Aleurocanthus spiniferus | 9 | Species |  |
| 6 | Pucciniomycetes | UK | Coleosporium asterum | 8 | Species |  |
| 8 | Insecta | UK | Liriomyza sativae | 6 | Species |  |
| 4 | Triplonchida | UK | Trichodorus cedarus | 5 | Species |  |
| 14 | Xanthomodales | UK | Xanthomonas arboricola | 5 | Species |  |
| 18 | Sordariomycetes | UK | Colletotrichum theobromicola | 5 | Species |  |
| 30 | Insecta | UK | Liriomyza huidobrensis | 5 | Species |  |
| 15 | Insecta | UK | Helicoverpa | 4 | Genus |  |
| 17 | Sordariomycetes | UK | Colletotrichum | 3 | Genus |  |
| 21 | Insecta | UK | Bemisia | 3 | Genus |  |
| 22 | Sordariomycetes | UK | Colletotrichum boninense | 3 | Species |  |
| 28 | Insecta | UK | Thaumetopoea pityocampa | 3 | Species |  |
| 36 | Viroids | UK | Potato Spindle Tuber Viroid | 3 | Species |  |
| 3 | Pseudomonadales | UK | Pseudomonas syringae pv. ulmi | 2 | Variant |  |
| 26 | Insecta | UK | Opogona sacchari | 2 | Species |  |
| 27 | Insecta | UK | Paysandisia archon | 2 | Species |  |
| 7 | Insecta | UK | Agromyzidae | 1 | Family |  |
| 10 | Insecta | UK | Thysanoptera | 1 | Order |  |
| 13 | Pucciniales | UK | Thekopsora minima | 1 | Species |  |
| 16 | Sordariomycetes | UK | Colletotrichum siamense | 1 | Species |  |
| 20 | Chromadorea | UK | Hirschmanniella caudacrena | 1 | Species |  |
| 23 | Peronosporea | UK | Phytophthora austrocedri | 1 | Species |  |
| 25 | Pucciniomycetes | UK | Melampsora ferrinii | 1 | Species |  |
| 29 | Xanthomodales | UK | Xanthomonas hydrangeae | 1 | Species |  |
| 31 | Insecta | UK | Noctuidae | 1 | Family |  |
| 32 | Insecta | UK | Spodoptera | 1 | Genus |  |
| 33 | Patatavirales | UK | Potato virus Y | 1 | Species |  |
| 34 | Insecta | UK | Rhynchophorus phoenicis | 1 | Species |  |
| 35 | Insecta | UK | Thrips | 1 | Genus |  |
